# The proteomic characterization of the peritumor microenvironment in human hepatocellular carcinoma

**DOI:** 10.1038/s41388-022-02264-3

**Published:** 2022-03-21

**Authors:** Yuhan Gu, Yuanyuan Guo, Na Gao, Yan Fang, Chen Xu, Guiming Hu, Mengxue Guo, Yaxing Ma, Yunfei Zhang, Jun Zhou, Yanlin Luo, Haifeng Zhang, Qiang Wen, Hailing Qiao

**Affiliations:** 1 Institute of Clinical Pharmacology, Zhengzhou University, Zhengzhou, China; 2 Department of Pharmacy, The First Affiliated Hospital of Zhengzhou University, Zhengzhou, China; 3 Affiliated Cancer Hospital of Zhengzhou University, Zhengzhou, China; 4 Affiliated People’s Hospital of Zhengzhou University, Zhengzhou, China

**Keywords:** Cancer microenvironment, Predictive markers, Prognostic markers

## Abstract

The tumor microenvironment (TME) was usually studied in tumor tissue and in relation to only tumor progression, with little involved in occurrence, recurrence and metastasis of tumor. Thus, a new concept “peritumor microenvironment (PME)” was proposed in the proteomic characterization of peritumor liver tissues in human hepatocellular carcinoma (HCC). The PME for occurrence (PME-O) and progression (PME-P) were almost totally different at proteome composition and function. Proteins for occurrence and progression rarely overlapped and crossed. Immunity played a central role in PME-O, whereas inflammation, angiogenesis and metabolism were critical in PME-P. Proteome profiling identified three PME subtypes with different features of HCC. Thymidine phosphorylase (TYMP) was validated as an antiangiogenic target in an orthotopic HCC mouse model. Overall, the proteomic characterization of the PME revealed that the entire processes of HCC occurrence and progression differ substantially. These findings could enable advances in cancer biology, diagnostics and therapeutics.

## Introduction

The tumor microenvironment (TME), which describes the composition of tumor tissue, plays an important role in tumorigenesis. The TME is usually studied in tumor tissue, and as such the involvement of the TME in processes associated with initial tumorigenesis and tumor recurrence as well as metastasis after surgery is unclear. Tumor cells and the tissues in which tumors grow are highly heterogeneous [1], as was first suggested in Paget’s “seed and soil” hypothesis [2]. The “soil” refers to nontumor tissue that provides a suitable environment for tumor cell growth, but is not itself tumor tissue. However, identifying and studying “the soil” in which tumor will grow in the future is difficult. Thus, a new concept, the peritumor microenvironment (PME), was developed to describe the mechanisms underlying the occurrence and progression of HCC. The PME includes tissue surrounding the tumor that is very close to the “soil”. The occurrence, recurrence and metastasis of tumor mainly depends on the PME, and thus both the PME and TME can influence tumor progression. To date, few studies have focused on the PME.

Hepatocellular carcinoma (HCC) is the most common form of liver cancer and is the sixth most-frequently diagnosed cancer and the fourth leading cause of cancer death worldwide [3]. In contrast to other types of tumors, more than 80% of HCC had adjacent tissues that showed cirrhosis or fibrosis [4]. Therefore, the PME in HCC may have unique characteristics. In this study, we carried out the first proteomic characterization of the PME in HCC using 41 and 71 samples from normal human liver tissues and HCC peritumor tissues, respectively.

## Result

### Proteomic analysis of the PME in patients with HCC

**Fig. 1:**
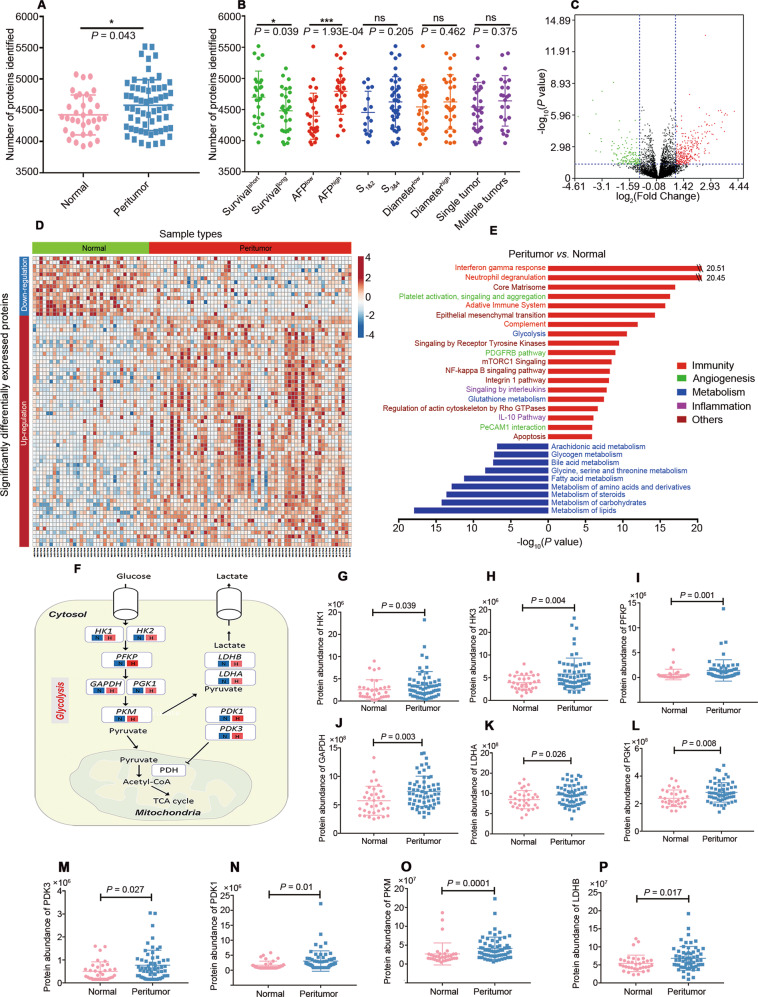
Proteomic analysis summary for the PME in patients with HCC. **A** Box plots of number of proteins identified in normal (pink, *n* = 34) and peritumor (blue, *n* = 61) liver tissues. **B** Comparison of the number of proteins identified in peritumor tissues grouped by survival time, AFP level, Cirrhosis stage, maximum tumor diameter and single or multiple tumors. Data are presented as the mean ± SD. **C** Volcano plot displaying differentially expressed proteins in the PME with *P* < 0.05 (Student’s *t* test). **D** Heatmap of significantly differentially expressed proteins. Each column represents one independent sample, and rows represents different proteins. Colors represent the protein expression level in the sample. **E** Signaling pathways involved by altered expression of proteins in peritumor group. The abscissa is -log10 (the *P* value enriched in the pathway). Red lines mean up-regulation, blue lines mean down-regulation. **F** Abnormal activation of glycolytic metabolic pathways (N, normal; H, peritumor), the shading represents the abundance of protein in each group. (**G–P**) Differential expression of key regulatory proteins involved in glycolytic metabolic pathways. Data are presented as the mean ± SD.

Across the entire data collection period, high stability and reproducibility were evidenced by high values for the interexperiment correlation coefficients (Fig. S1A–E). A total of 6 947 proteins were identified in the PME and the subcellular distribution of these proteins was annotated with Gene Ontology (Fig. S1F). The average number of proteins identified in the PME (4543) was significantly higher than that for normal liver tissues (4 372) (Fig. 1A). Furthermore, patients with high levels of α-fetoprotein (AFP^high^; AFP > 300 ng/mL) and a short survival time (survival^short^; survival time <344 days) had a higher number of identified proteins in the PME (Fig. 1B). These results suggested that changes in protein expression in the PME were closely connected with clinical indicators of HCC and that aberrant increases in the number of proteins may be related to HCC progression.

Comparison of PME and the control revealed 1360 differentially expressed proteins (Fig. 1C, D). Enrichment analysis demonstrated that the differentially expressed proteins in the PME were associated with signaling pathways that may be related to HCC occurrence and progression (Fig. 1E). Notably, expression of key regulatory proteins in the glycolysis pathway was significantly upregulated (Fig. 1F–P), indicating that hepatic-specific metabolic pathways are reprogrammed in the PME of HCC.

### PME for occurrence (PME-O)

**Fig. 2:**
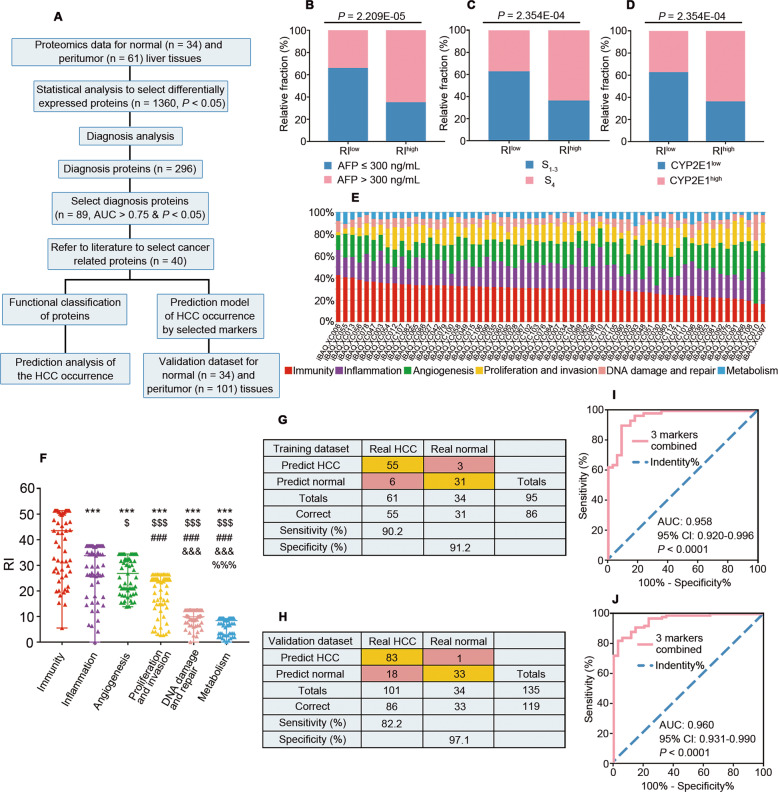
Predictive assessment of risk factors for the occurrence of HCC. **A** Workflow of the relationship between the altered PME proteome and HCC occurrence. Differential proportion of distribution for AFP (**B**), cirrhosis stage (**C**) and CYP2E1 activity (**D**) between RI^low^ and RI^high^ groups divided by the median RI. Detailed workflow of calculations for RIs described as Fig. S2A. RI^high^, RI > 136.17. CYP2E1^high^; CYP2E1 > 1 350 pmol/min/mg. **E** Proportion of risk factors for HCC occurrence in each patient. **F** Comparison of the RI among different risk factors related with HCC occurrence; Data are presented as a scatter diagram representing the median with range. ROC curve (**I**, **J**) and sensitivity as well as specificity (**G**, **H**) of the HCC occurrence prediction model with three markers combined (HSPA4L, VIL1, TYMP) in our dataset (**G**, **I**) and the validation dataset (**H**, **J**). ^***^P < 0.001 compared with immunity; ^$^*P* < 0.05, ^$$$^*P* < 0.001 compared with inflammation; ^###^*P* < 0.001 compared with angiogenesis; ^&&&^*P* < 0.001 compared with proliferation and invasion; ^%%%^*P* < 0.001 compared with DNA damage and repair. RI, risk index.

Unlike traditional protein analysis methods that focus on a single protein or a single factor such as immunity [5, 6], inflammation [7, 8], angiogenesis [9], or metabolism [10], in this study we established a method to examine multiple integrated factors (Fig. 2A). Briefly, risk indices (RIs) calculated for all 40 proteins identified in the PME-O (Table S3) had values ranging from 32.40 to 169.64 (interquartile range [IQR], 113.75~154.68; average, 127.73; Fig. S2B). The RIs correlated well with AFP, cirrhosis stage, and CYP2E1 activity (Fig. 2B–D). Patients with high levels of RI (RI^high^, RI > 136.17) were more likely to have higher AFP level and cirrhosis stage as well as CYP2E1 activity, indicating that the established method has substantial clinical value.

Using the functional classification and method described above, we found that the proportion of several factors varied substantially among patients with HCC (Fig. 2E), and this variation could be attributed to high levels of heterogeneity. The RI for immunity was 43.56, which was highest among the six factors, followed by inflammation (33.78) and angiogenesis (26.82) (Fig. 2F). In general, immunity was commonly the leading cause of HCC occurrence in the PME.

To facilitate the clinical application of this information, the abovementioned 40 proteins were further optimized for screening. Using a logistic regression method, we constructed an occurrence prediction model that considered three protein markers (immunity protein, HSPA4L; inflammation protein, VIL1; and angiogenesis protein, TYMP) that were selected according to the AUC and weight (Fig. S2C–H). The model yielded a sensitivity of 90.2% and a specificity of 91.2% for HCC in our dataset (Fig. 2G) and a sensitivity of 82.2% and specificity of 97.1% in another dataset (Fig. 2H). We also demonstrated that this model could differentiate HCC from normal in both our data set (AUC = 0.958) and the validation dataset (AUC = 0.960; Fig. 2I, J), suggesting that the model we established based on findings for PME in this study could have high value for predicting the risk of HCC occurrence.

### PME for progression (PME-P)

**Fig. 3:**
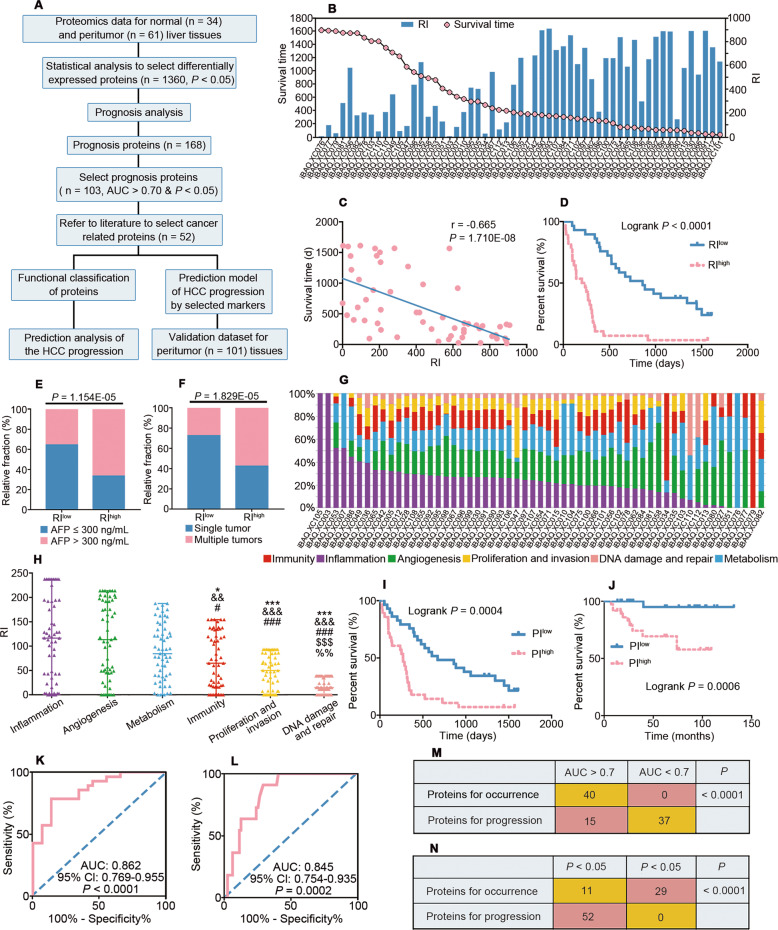
Predictive assessment of risk factors for the progression of HCC. **A** Workflow of the relationship between the altered proteome of the PME and HCC progression. **B** Distribution of RI and survival time for each HCC patient. **C** Correlation between the RI and survival time. **D** Kaplan–Meier curve analysis based on the RI value grouped by the median. Differential proportion of distribution for AFP (**E**) and single or multiple tumors (**F**) according to different RI levels (f, RI^high^, RI > 484.18; g, RI^high^, RI > 437.32). **G** Proportion of risk factors for the progression of HCC in each patient. Detailed workflow of calculations for RIs described as Fig. S3A. **H** Comparison of the RI among different risk factors related with HCC progression. Data are presented as the median with range. ^*^P < 0.05, ^***^P < 0.001 compared with inflammation; ^&&^*P* < 0.01, ^&&&^*P* < 0.001 compared with angiogenesis; ^#^*P* < 0.05, ^###^*P* < 0.001 compared with metabolism; ^$$$^*P* < 0.001 compared with immunity; ^%%^*P* < 0.01 compared with proliferation and invasion. RI, risk index. Overall survival curves (**I**, **J**) and ROC curves (**K**, **L**) of the HCC progression prediction model with three markers combined (CMPK2, TYMP, NADSYN1) according to the median PI in our dataset (**I**, **K**) and in the validation dataset (**J**, **L**). PI, prognostic index. Detailed calculations for PI described in METHODS section. **M** Comparison of the proteins for occurrence and progression in predicting HCC occurrence. Number of proteins was counted according to AUC of ROC curve analysis. *P* value of four-fold table was calculated by Chi-square test and Fisher exact tests. **N** Comparison of the proteins for occurrence and progression in predicting HCC progression. Number of proteins was counted according to Kaplan-Meier survival curve analysis and log-rank test. *P* value of four-fold table was calculated by Chi-square test and Fisher exact tests.

Further analysis of the correlation between protein function and disease progression identified 52 proteins in the PME-P that could be divided into six categories by function (Table S4; Fig. 3A). We then used RIs to test the influence of various factors on progression and individual variations. The calculated RIs for these 52 proteins (Table S5) ranged from 0.26 to 908.65 (IQR, 172.15~705.89; average, 447.10; Fig. S3B). Similar to those in the PME-O, patients with RI^high^ were more likely to have shorter survival time and higher AFP level and multiple tumors rather than single tumor (Fig. 3B–F).

Based on the above method, we calculated the proportion of risk factors related to progression for each patient (Fig. 3G). The average RIs for inflammation, angiogenesis and metabolism were 116.45, 113.44, and 84.43, respectively, which were much higher than those for the other factors, but the difference between the three was not significant (Fig. 3H). Several factors including immunity, inflammation, angiogenesis, metabolism, proliferation and invasion have been shown to influence the progression of cancer [11]. Overall, in this study, the progression of HCC in each patient was determined by different factors, with inflammation, angiogenesis and metabolism being the main influencing factors of HCC progression in the PME, and these three factors all contributed to HCC progression.

We constructed a progression prediction model involving three markers (inflammation protein, CMPK2; angiogenesis protein, TYMP; and metabolism protein, NADSYN1) that superior to a single protein (Fig. S3C-I). The median survival time in the RI^low^ group was significantly longer than that in the RI^high^ group by a log-rank test (*P* = 0.0004 in our dataset and *P* = 0.0006 in the validation dataset; Fig. 3I, J). Meanwhile, ROC analysis showed that this model could better predict disease progression [our dataset: AUC, 0.862; and 95% CI, 0.769 to 0.955; validation dataset: AUC, 0.845; 95% CI, 0.754 to 0.935] (Fig. 3K, L), suggesting that the model we built based on the findings for PME in this study could be valuable for predicting the risk of HCC progression.

### Signatures for the PME subtypes of HCC

The PME-O and PME-P, composed of 40 and 52 proteins that were highly correlated with disease occurrence and progression, respectively, constituted the HCC PME. Notably, only 3.37% (3/89) of proteins were involved in both disease occurrence and progression. To further verify the difference, we have evaluated the effect of the proteins for occurrence and progression on occurrence and progression, respectively (Fig. 3M, N). We found that proteins for progression have only a weak effect on occurrence of HCC, and occurrence proteins have only a weak effect on progression similarly. The proteins for occurrence and progression were different at composition and function, which means occurrence and progression of HCC were totally different stages, implying that different strategies for prevention and treatment are needed. In addition, clinical factors, and individual variations were all reflected by the large difference in the occurrence and progression of HCC, which further highlighted the high levels of heterogeneity in the PME that underpinned the stratification analysis.

**Fig. 4:**
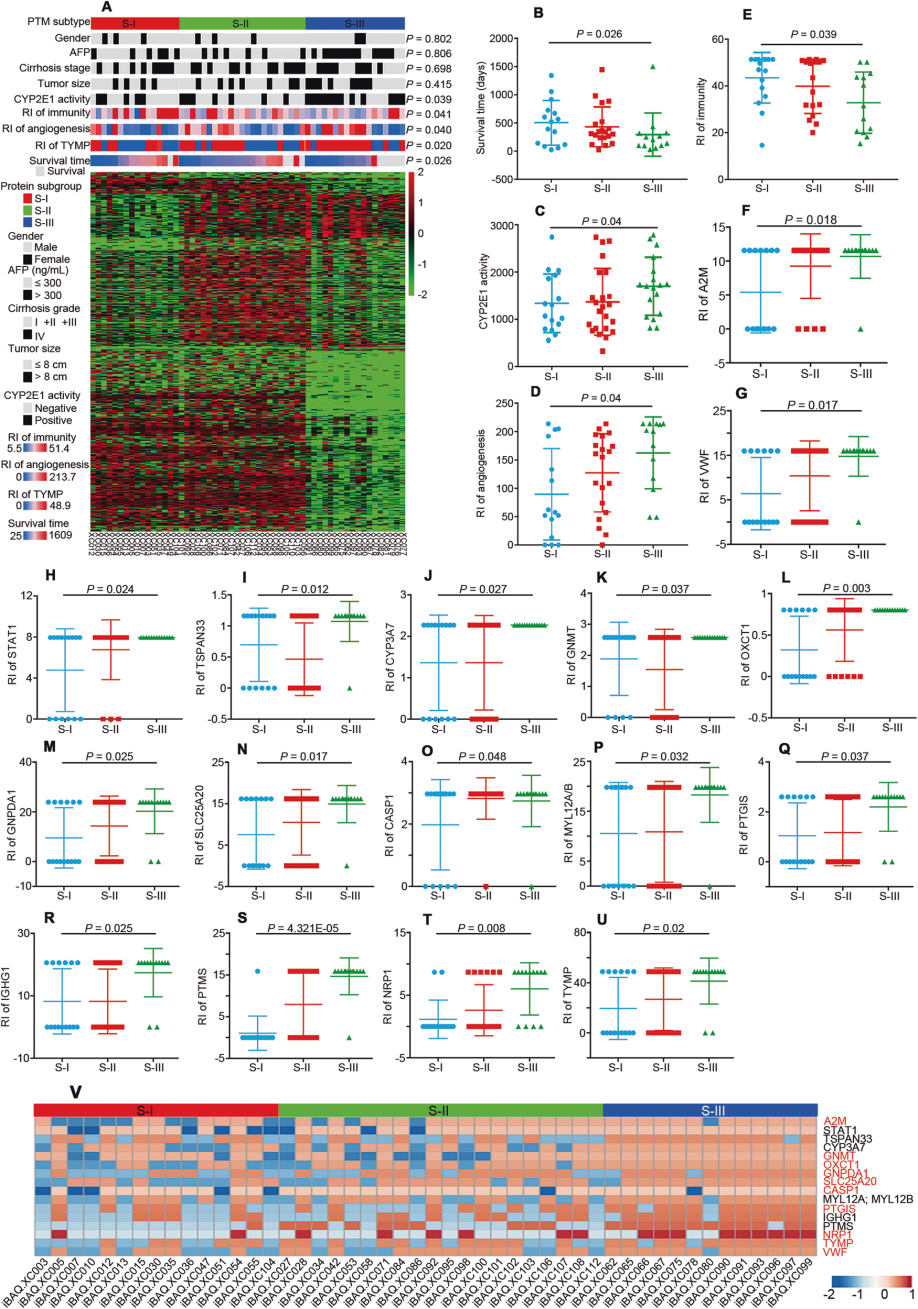
Molecular typing based on HCC PME. **A** Each column represents a patient sample and rows indicate proteins. The color of each cell shows the relative protein abundance (log_2_-transformed). The proteomic subtypes are annotated on the top of the heatmap by colored bars (S-I: red; S-II: green; and S-III: blue). Comparison of survival time (**B**), CYP2E1 activity (**C**), RI of angiogenesis (**D**) and RI of immunity (**E**) among three molecular subtypes. (**F–U**) Comparison of RI for 16 proteins with significant differences among the three molecular subtypes. Data are presented as the mean ± SD. **V** Heatmap of the RI for 16 proteins with significant differences and 10 potential drug targets (red font) for the three molecular subtypes. TYMP, thymidine phosphorylase. RI, risk index.

In this study, tumor classification was, for the first time, based on the PME rather than the TME and thus could consider the entire process of occurrence and progression. Using consensus clustering analysis, three proteomic subtypes were clearly identified based on differentially expressed proteins (Fig. S4). All the cases were divided into three subtypes and 18, 23 and 16 cases were classified as subtypes S-I, S-II and S-III, respectively (Fig. 4A). Notably, the overall survival (OS) of 57 patients was reflected by a median follow-up time of 344 days (range: 25–1609 days), and among the S-I, S-II and S-III subtypes the average follow-up times were 504, 432, 293 days, respectively (Fig. 4B).

In terms of molecular characteristics of HCC, the subtypes S-I, S-II and S-III showed significant differences in immunity for PME-O (43.49, 39.86, and 32.86) and angiogenesis for PME-P (89.51, 127.19, and 162.31) (Fig. 4C, D). Significant differences among the three subtypes were also found for CYP2E1 activity (1 341.44 pmol/min/mg protein, 1 370.02 pmol/min/mg protein, and 1 700.58 pmol/min/mg protein) (Fig. 4E). Moreover, there were significant differences in the RIs of proteins that were specific to molecular subtypes.

According to the PME, 38 proteins could be targeted by drugs approved by the U.S. Food and Drug Administration (FDA) or candidate drugs that are currently in clinical trials as listed in the DrugBank database (Table S7). Of the 16 proteins with significant differences at RI among the three subtypes (Fig. 4F–U), 10 proteins could be considered as potential targets (Fig. 4V), suggesting that the characteristics of the three subtypes in the PME are obviously different and thus would require different treatment strategies.

### TYMP as a potential target for the HCC

**Fig. 5:**
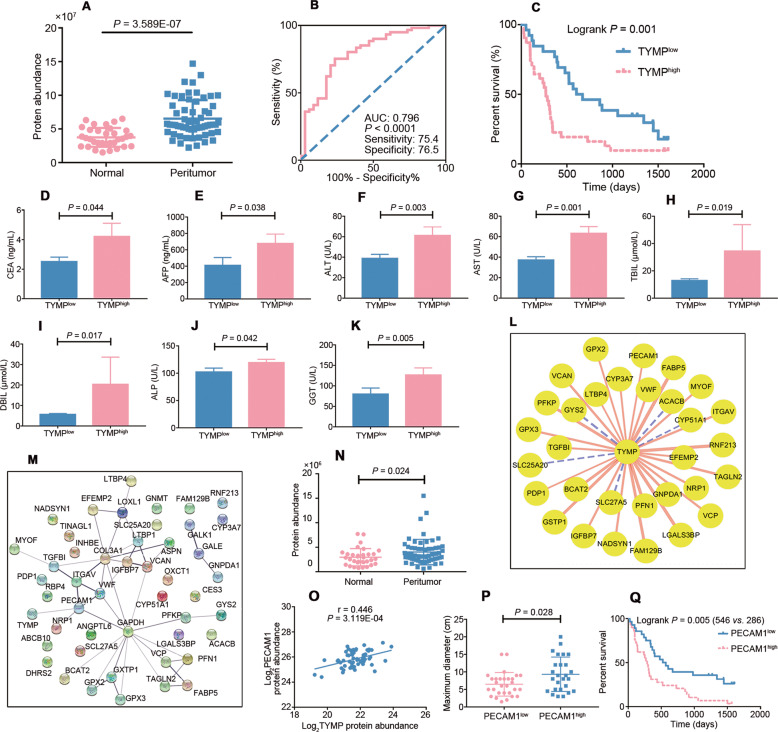
Relationship between TYMP expression and clinical parameters and screening for interacting proteins. Quantification of TYMP levels in normal and peritumor samples in our proteomic data (**A**). (**B**, **C**) Relationship between TYMP expression and the occurrence and progression of HCC in our dataset. (**D–K**) Relationship between TYMP expression and serum level of liver function index. **L** Proteins significantly associated with TYMP in the microenvironmental proteome of HCC. **M** STRING database prediction of potential interaction between TYMP and PECAM1. **N** Significant increase in PECAM1 expression in peritumor tissue in our data set. **O** Positive correlation between expression of TYMP and PECAM1 in our data set. Data are presented as the mean ± SD. (**P, Q**) Relationship between PECAM1 expression and maximum diameter as well as survival time.

Our samples exhibited higher abundance of TYMP in the PME (Fig. 5A), while the expression of TYMP mRNA did not differ significantly between the two groups (Fig. S5A). Consistent with proteomic data, the expression of TYMP protein detected by Western blot is elevated in peritumor group (Fig. S5B). TYMP expression was significantly correlated with diagnosis (Fig. 5B) and prognosis (Fig. 5C) as well as clinical parameters (Fig. 5D–K). Furthermore, high TYMP expression levels and the effect on occurrence and progression were consistent with listing in the PRIDE database (Fig. S5C–E), The Cancer Genome Atlas (TCGA) data (Fig. S5F) and with our proteomic data. In addition, we found that TYMP significantly correlated with PECAM1 (Fig. 5L–O; Fig. S5G–I), an indicator of microvascular density that was up-regulated in the PME, and positively correlated with tumor size and prognosis (Fig. 5P–Q).

**Fig. 6:**
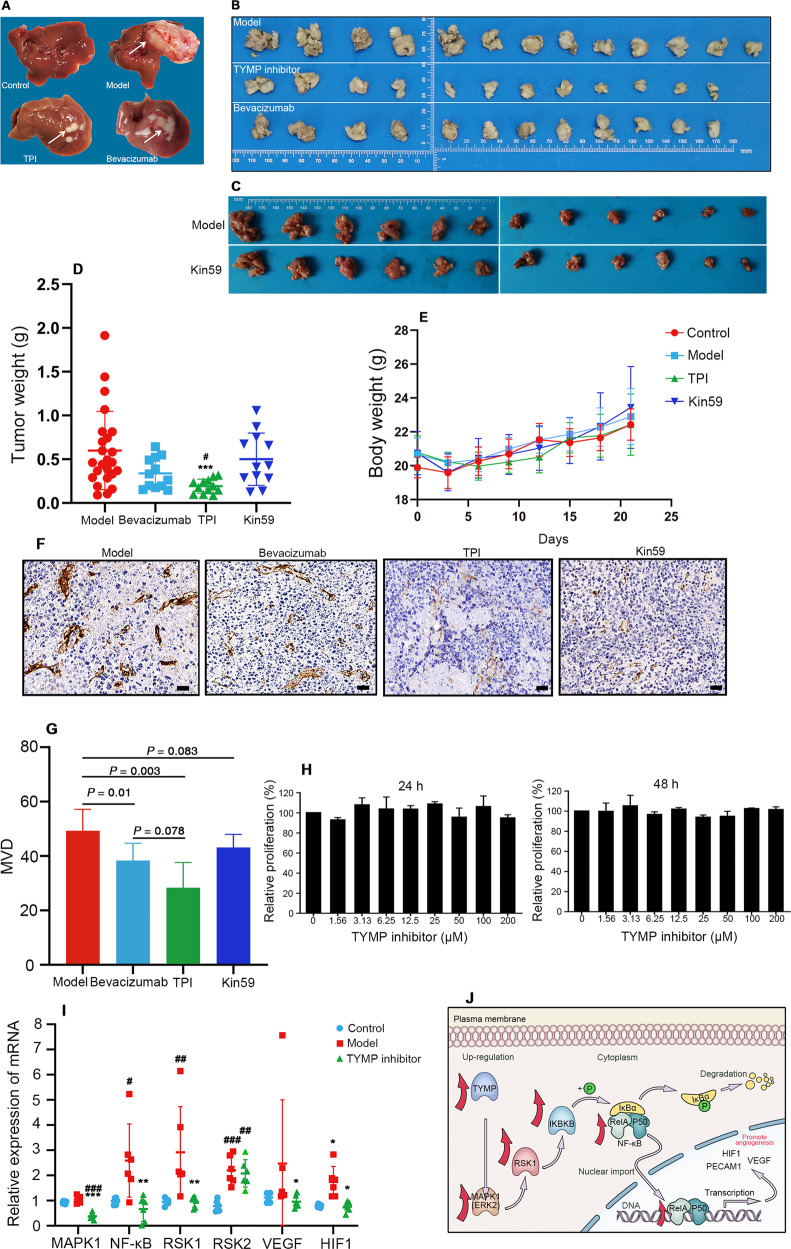
TYMP is a potential antiangiogenic target for HCC. **A–C** Action of TPI (100 mg/kg) and Kin59 (30 mg/kg) in a liver orthotopic transplantation tumor model with H22 cell lines in BALB/c mice. Body weight (**D**) and Tumor weight (**E**) of BALB/c mice in different group. Data are presented as the mean ± SD. ^***^P < 0.001 compared with normal; ^#^*P* < 0.05 compared with bevacizumab group. **F** Representative immunostaining images of PECAM1 (CD31), marker of microvascular density in tumor tissues for each group (Scale bar: 50 μm). **G** microvascular density analysis of PECAM1 (CD31) immunostaining, data are presented as the mean ± SD. **H** Effect of TPI on proliferation of H22 mouse liver cancer cells after treatment for 24 h and 48 h. **I** Expression of mRNA of TYMP related proteins in different groups, data are presented as the mean ± SD. ^*^P < 0.05, ^**^P < 0.01, ^***^P < 0.001 compared with model; ^#^*P* < 0.05, ^##^*P* < 0.01, ^###^*P* < 0.001 compared with normal^.^ **J** Schematic of the mechanism underlying the upregulation of TYMP expression in angiogenesis.

Tipiracil (TPI) is one of the most promising TYMP inhibitors, which is used to prevents rapid degradation of trifluorothymidine (TFT) in an orally administered fixed-dose formulation TAS-102 [12, 13]. TAS-102 is approved for metastatic colorectal and gastric cancer, while the antiangiogenic effect of TPI has not been reported in HCC. Another TYMP inhibitor 5′-O-Trityl-inosine (Kin59) [14] also shows antiangiogenic effect in vitro [15], but there is a lack of anti-tumor effect of kin59 in vivo. Considering the immunity is an important factor in peritumor microenvironment, H22 mouse HCC cell line was used in the transplanted BALB/c mice as it represents a syngeneic model with animals having an intact immune system. In this study, the two TYMP inhibitors treatment reduced tumor weight (Fig. 6A–D) to varying degrees, but they have no significant effect on body weight (Fig. 6E). The intratumoral neovascularization was also reduced by the two TYMP inhibitors (Fig. 6F, G). Apparently, the effect of tumor suppression is obviously related to the antiangiogenesis ability of TYMP inhibitor. Consistent with strong effect of inhibition of intratumoral neovascularization, TPI treatment remarkably reduced tumor weight. The effect of antiangiogenesis of Kin59 was inferior than TPI, thus showing a weaker anti-tumor effect. The ability of inhibiting neovascularization of TYMP inhibitor may determine the tumor suppression effect. Importantly, the inhibitory effect of TPI was superior to that of bevacizumab, an antiangiogenic drug that has been used in the clinic. In addition, TPI had no significant effect on the growth and proliferation of H22 cells in vitro at 24 h and 48 h, respectively, suggesting that this inhibitor targets the tumor microenvironment rather than tumor cells (Fig. 6H). Collectively, these data provide evidence that TYMP could be used as an antiangiogenic target for HCC.

To examine the mechanism of TYMP in HCC angiogenesis, we analyzed two common and distinct signaling pathways in angiogenesis (Fig. S6A–I). The expression level of proteins involved in the MAPK1 (ERK2)/RSK1/NF-κB pathway was significantly up-regulated (Fig. S6F–I), and the elevated protein abundance was associated with TYMP expression level (Fig. S6J–M). With the treatment of TYMP inhibitor, the mRNA of MAPK1 (ERK2)/RSK1/NF-κB pathway was down-regulated, consistent with common angiogenesis-related protein VEGF and HIF1 (Fig. 6I), indicating that TYMP upregulation promoted neovascularization through the MAPK1 (ERK2)/RSK1/NF-κB pathway (Fig. 6J). Taken together, these findings suggest that TYMP is a potential target for HCC.

## Discussion

Comprehensive peritumoral proteomic analysis has led to a deeper understanding of the occurrence and progression of HCC. Herein, we proposed a new concept of the PME, which provided new insights into the whole process of HCC, from occurrence to progression. Previous studies focusing on the TME could only explore progression and not the occurrence of HCC [16, 17]. As we all know, protein rather than mRNA is the performer of various physiological and pathological functions. Therefore, the abundance of protein reflects the state of the disease more than the expression of mRNA. Previous studies found protein and RNA have different enrichment patterns across tissues, which may be caused by post-translational regulation, different turnover rate at RNA and protein levels [18]. In this study, we found the elevated TYMP protein expression, while the expression of TYMP mRNA did not differ significantly between the two groups. Our research provided different perspectives to reveal undiscovered changes in RNA-seq (mRNA) approaches. Based on proteomics data of peritumoral tissues, the PME was not only related to occurrence, recurrence and metastasis but was also related to the progression of HCC.

According to proteomic characterization, the PME was divided into the PME-O and PME-P, which represented the characterization of occurrence and progression, respectively. Unlike traditional protein analysis methods that focus on single proteins or single factors, in this study, we established a method to examine multiple integrated factors, such as immunity [5, 6], inflammation [7, 8], angiogenesis [9, 19], metabolism [10], etc. In the proteomic composition, the PME consisted of 89 proteins, among which 40 proteins were related to the occurrence of HCC, 52 proteins were related to progression, and only 3.37% of the proteins in the PME were shared by both occurrence and progression. Functionally, the PME-O had an impact on occurrence, recurrence and metastasis, and both the PME and the TME influenced the progression of tumors. Moreover, the proteomic characterization of the PME-O and PME-P significantly differed; immunity played a central role in the PME-O, while inflammation, angiogenesis and metabolism were key factors that influenced the PME-P. These findings explained why most studies [20–23] showed that tumor occurrence was the result of tumor cells escaping the immune response. Moreover, only a few proteins participated in both occurrence and progression, indicating that the processes of occurrence and progression are totally different, which could further explain why the process of occurrence may happen sooner or last longer and the speed of progression may be slower or faster among different individuals. Based on the PME-O and PME-P, two models were successfully built to accurately predict HCC occurrence and progression, respectively.

Proteome profiling identified three PME subtypes of HCC based solely on the altered proteome. Tumor molecular subtyping is a new classification system based on the molecular characteristics of tumor tissues, which is different from the traditional pathological classification system. All previous studies performed molecular typing based on the TME [24–27], which meant that only tumor progression was described. In this study, for the first time the tumor classification was based on the PME not the TME and thus could include the entire process of occurrence and progression. In this study, these three PME subtypes of HCC accurately reflected multiple features of HCC, such as survival time, potential drug targets, CYP2E1 activity, immunity and angiogenesis, supporting the superior predictive power of our proteomic clustering. It is worth mentioning that CYP2E1 has been shown to be causally related to hepatocarcinogenesis in previous studies [28, 29], which further verified the significance and value of PME subtypes.

Additionally, according to the PME, a total of 38 potential targets were identified, among which TYMP, also known as platelet endothelial cell growth factor (PD-ECGF, ECGF1) [30], was of great interest. TYMP participates in nucleic acid metabolism under physiological conditions and inversely catalyzes thymine and 2-D-deoxyribose-1-phosphate [31]. Clinical findings have shown that TYMP is a marker that reflects the characteristics of the tumor stroma [32, 33] and is closely related to poor prognosis in various cancers [34–36]. However, the role and related mechanism of TYMP in the occurrence and progression of HCC are still unclear. In this study, we demonstrated that TYMP played a protumor role by affecting angiogenesis through the MAPK1 (ERK2)/RASK1/NF-κB signaling pathway. Importantly, the inhibitory effect of TPI on an H22 orthotopic tumor model was superior to that of bevacizumab in clinical applications. Although the antiangiogenesis effect of Kin59 is not as significant as that of TPI, it still showed anti-tumor effect, which may be related to the effect of TYMP inhibition of Kin59. Compared to TPI (IC_50_ = 0.014 ± 0.002 μM) [37], Kin59 (IC_50_ = 30 ± 15 μM) [38] has a weaker inhibitory effect on TYMP. The antiangiogenesis effect of TYMP is obviously related to the inhibitory effect, and may determine the inhibitory effect on tumors. Besides, TYMP knockout or inhibition also showed no effect on coagulation and bleeding [39]. According to known studies, no obvious toxicity of target TYMP has been found [40]. In this study, the two TYMP inhibitors have no significant effect on body weight. Based on the above results, TYMP is an effective and safety anti-HCC target.

In summary, in this study we present a new concept, the PME, which is based on proteomic characterization of human HCC. The PME involves the entire process of HCC occurrence and progression, in contrast to TME, which is related only to progression. Moreover, our results showed that the processes of occurrence and progression differed substantially in terms of proteome composition and function. Proteins for progression have only a weak effect on occurrence of HCC, and occurrence proteins have only a weak effect on progression similarly, suggesting that different strategies for HCC prevention and treatment are needed at different stages of disease. We propose a new classification method for HCC that is based on the PME. This classification could more accurately reflect multiple features of HCC and information for the PME in HCC could facilitate new advances in cancer. Overall, the new developed knowledges for the PME will enable new advances in cancer biology, diagnostics and therapeutics.

## Methods

### Liver tissue collection

Liver tissues were collected from Affiliated People’s Hospital of Zhengzhou University (Zhengzhou, China) and Affiliated Cancer Hospital of Zhengzhou University (Zhengzhou, China). The study protocol was approved by the ethics committee of Zhengzhou University. Written informed consent was obtained from each patient. (Detail in Supplement methods).

### Proteomic processing of liver tissue

Liver protein was extracted and digested. The BCA method (Boster Biological Technology, Wuhan, China) was used to determine protein concentrations [25, 41]. A peptide solution produced from HEK293T cells was used as a quality control standard (method reference [27]). Peptides were prefractionated by High-pH reverse-phase and analyzed using liquid chromatography-mass spectrometry-tandem mass spectrometry (Q-Exactive HF LC-MS/MS). (Detail in Supplement methods).

### Proteomic processing of raw data

Raw data was Identification and quantification of protein by MaxQuant software (version 1.5.3.8) [42]. Intensity-based absolute protein quantification (iBAQ) [43] based on peak intensity was used to express protein expression levels. The R/Bioconductor package limma v.3.24.15 was used to apply the normalized quantile function to normalize expression matrix quantiles [44, 45]. (Detail in Supplement methods).

### Protein alterations in the PME

The R/Bioconductor package limma v.3.24.15 [46] was used to identify differentially expressed proteins between normal liver tissues and peritumor tissues. Proteins detected in <50% of samples were excluded, and missing values were replaced with half the minimal value for each protein. Differences greater than 1.2 and *P* < 0.05 were considered significant.

### Proteomic subtype identification

Non-negative matrix factorization (NMF) [47] is frequently used in high-throughput biological experiments [48]. In this study, the non-negative matrix decomposition consistency clustering algorithm in the R language NMF software package (version 0.20.6) was used to perform cluster analysis on the proteome data, and molecular subtypes of the peritumor proteome were obtained. The CDF was used to estimate the efficacy of proteomic subtypes. A Chi-square test was used to examine the relationship between molecular subtypes and clinical characteristics.

### Changes in levels of proteins involved signaling pathways in the PME

Metascape software [49] (http://metascape.org/) was used to conduct pathway enrichment analysis of the differential proteome of peritumor tissue. The signal pathway databases used in the analysis include the Kyoto Encyclopedia of Genes and Genomes (KEGG) database [50], Hallmark Gene Sets [51], the Reactome Gene database [52], the Canonical Pathways [53] and BioCarta Gene Sets [53], for a total of 1 524 signaling pathways. Altered pathways were then annotated based on consultation of the literature.

### Proteomic characterization of the PME-O and PME-P

Receiver Characteristic Operator (ROC) curve analysis [46] and Kaplan-Meier survival curve analysis was used to analyze the differential proteome (*n* = 1360) of the PME. RI of each protein was calculated and proteomics data from the PRoteomics IDEntification (PRIDE) database [10] (www.ebi.ac.uk/pride/archive, accession numbers PXD006512) were downloaded for verification. The risk factors for the occurrence and progression of HCC was compared. (Detail in Supplement methods).

### Drug target discovery and verification based on the PME

#### Potential drug targets in the PME proteome

The DrugBank database [54] (https://www.drugbank.ca/) was searched for potential drug targets in the PME.

#### Detection and verification of TYMP expression

The iBAQ value of the protein TYMP was regarded as the protein expression abundance. The protein TYMP abundance in our data and data from the PRIDE database (accession number PXD006512) were used to compare the expression in different groups. The LIHC data in TCGA dataset and corresponding clinical data was downloaded from The Cancer Genome Atlas Program (https://www.cancer.gov/about-nci/organization/ccg/research/structural-genomics/tcga). The STRING database [55] (https://string-db.org/cgi/input.pl) was used to explore the proteins related to TYMP.

#### Liver orthotopic transplantation tumor model with H22 cell lines in mice

Male BALB/c mice was divided into 4 groups: the sham operation group, model group, TPI group, Kin59 group and bevacizumab group. The intervention group was given 100 mg/kg tipiracil and 30 mg/kg kin59 (in 20% DMSO, 20% cremophore in PBS) [39] respectively. The operations detail in Supplement methods.

#### Immunohistochemistry assay

The liver tissues were embedded in paraffin and sliced. The sample sections were deparaffinized and hydrated. After antigen retrieval, endogenous peroxidase was neutralized. ovine serum albumin was used to block antigen. The sections were incubated with CD31 primary (Abcam Cat# ab182981, Cambridgeshire, UK) and secondary antibody (Servicebio Cat: GB23303, Wuhan, China), the diaminobenzidine solution was added into the sections. After stained with hematoxylin, the sections were dehydrated and covered. The positive protein expression was examined by XSP-C204 (CIC, Beijing, China) and the images were analyzed by Image-Pro Plus 6.0.

Three high angiogenesis fields of each sample were chosen, and MVD (CD31) were calculated as numbers per field. The average number of three fields were considered as MVD of each sample. The operator was blinded to the group of sections.

### MTT assay

H22 cells were cultured with indicated concentrations of tipiracil (1.56, 3.13, 6.25, 12.5, 25, 50, 100 and 200 μM) for 24 h and 48 h. Then 3-(4,5-dimethylthiazol-2-yl)−2,5-diphenyltetrazolium bromide (MTT) (Solarbio Science & Technology, Beijing, China) was added and incubated for 4 h. The medium was discarded and DMSO was added. Formazan precipitate at 490 nm was detected using a microplate spectrophotometer.

### CYP2E1 activity determination

Human liver microsomes were prepared by hypothermal differential centrifugation [29]. The concentration of microsomal protein was determined by the Bradford method [56]. The CYP2E1 activity was determined according to the reference [57–59].

### RT-qPCR assay

Total RNA was extracted with Trizol (Vazyme Cat: R401-01-AA, Jiangsu, China) according to the manufacturer’s protocol. HiScript^®^ III All-in-one RT SuperMix kit (Vazyme Cat: R333-01, Jiangsu, China) was used to reverse transcription reaction. Taq Pro Universal SYBR qPCR Master Mix kit (Vazyme Cat: Q712-02, Nanjing, China) was used to real-time PCR. Primer sequences were listed in Supplementary Table S8.

For VEGF and RSK1, Mann–Whitney U test was used to calculate the significance of the difference between groups. For MAPK, NF-κB, RSK2 and HIF1, One-Way ANOVA test was used to calculate the significance.

### Western blotting

Liver samples were homogenated in RIPA lysate buffer (Solarbio Science & Technology R0010, Beijing, China) with PMSF, and protein was extracted according to the manufacturer’s protocol. Protein concentration was deter-mined by BCA Protein Assay Kit (GLPBIO GK10009, Cat: California, USA). Polyacrylamide gel is prepared according to the manufacturer’s protocol of PAGE Gel Fast Preparation Kit (Epizyme Biotech Cat: PG212, Shanghai, China). After electrophoresis (Bio-rad, California, USA), protein was transferred to a PVDF membrane. The PVDF membrane is blocked in defatted milk powder dissolved in TBST. Then the membrane was incubated with corresponding primary antibodies and secondary antibodies. The antibodies and diluted concentration used were as follows: anti-TYMP 1:1000 (Proteintech Cat: 12383-1-AP, Illinois, USA), anti-β-actin 1:5000 (Servicebio GB11001, Wuhan, China), and goat anti-rabbit IgG 1:10,000 (SAB Cat: L3012, Maryland, USA).

### Statistical analysis

SPSS 21.0 was used for statistical analysis. GraphPad Prism 7.0 and Cytoscape 3.7.0 software were used for graphing. Statistical tests included, but were not limited to, Student’s *t* test, the Shapiro–Wilk test, Kruskal–Wallis test, Chi-square test, and log-rank test. The variance of each group has been compared. Use parametric test for data with similar variance, and use non-parametric test for data with heterogeneity of variance. All analyses used two-sided tests, and *P* < 0.05 was considered statistically significant.

## Supplementary information

Supplement information

Supplement Tables

## Data Availability

The data that support the findings of this study are available within the paper and its Supplementary Information. The data files of proteome are available via ProteomeXchange with identifier PXD023118 (https://www.ebi.ac.uk/pride/, accession number PXD023118).
