# Supplementary material for: The proteomic characterization of the peritumor microenvironment in human hepatocellular carcinoma
Source: Oncogene. 2022 Mar 21;41(17):2480–91. doi: 10.1038/s41388-022-02264-3 (PMC9033583; doi:10.1038/s41388-022-02264-3)
Supplement: Supplementary file 1 — Supplement information [file 41388_2022_2264_MOESM1_ESM.pdf]

Fig. S1 Workflow of sample processing, quality control.

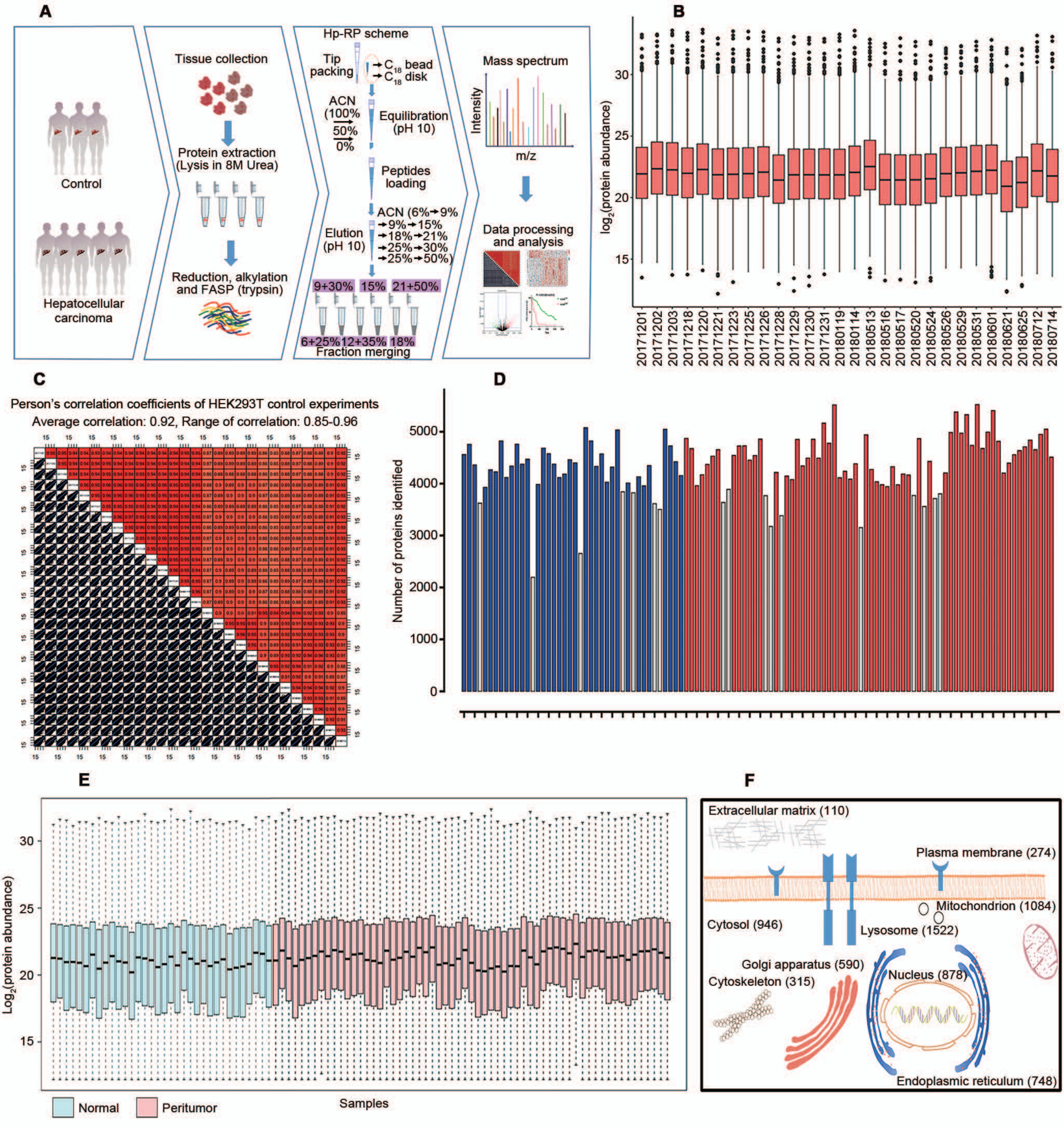

Fig. S2 Establishment of prediction scores and a prediction model of HCC occurrence.

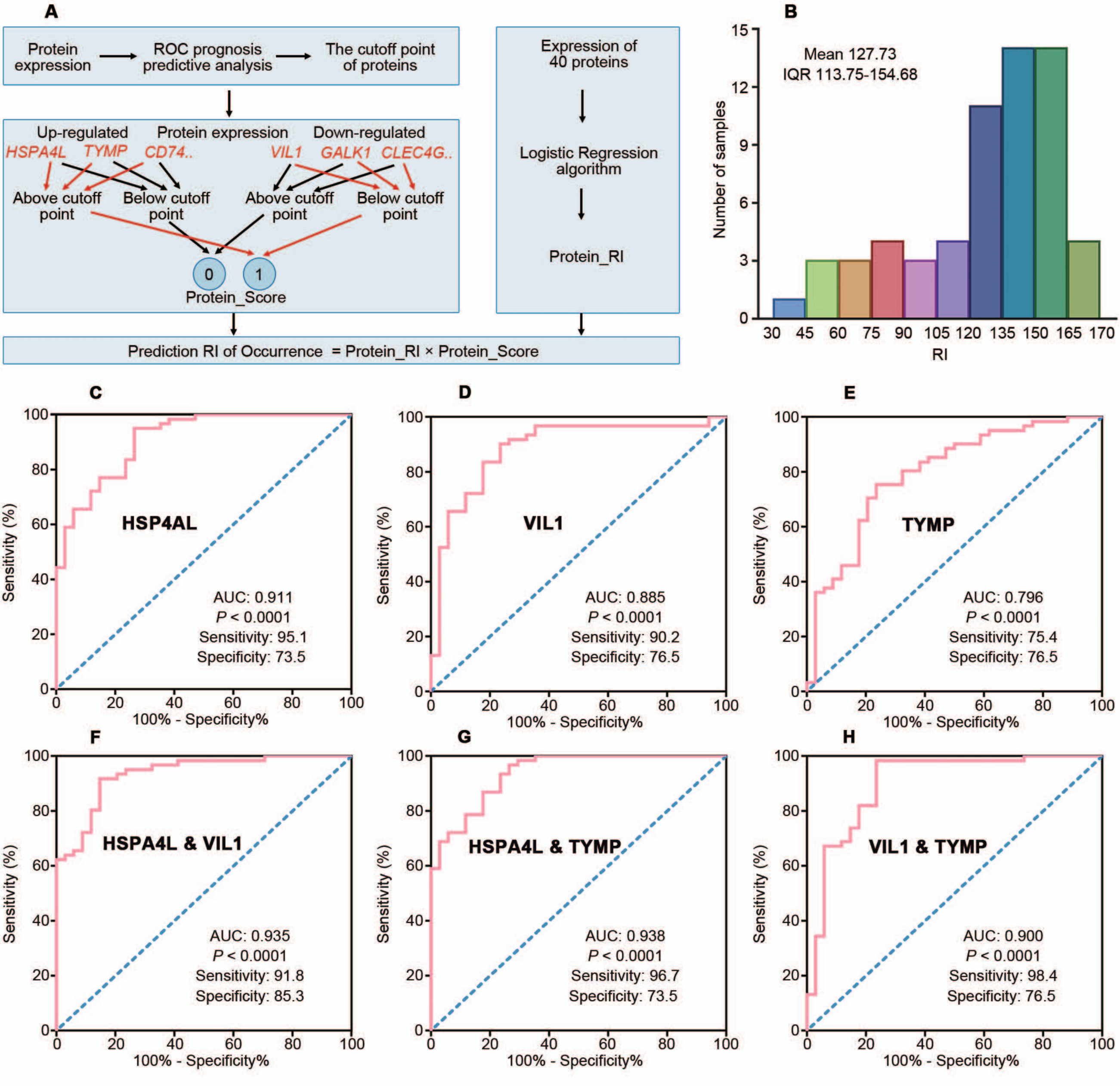

Fig. S3 Establishment of prediction scores and a prediction model of HCC progression.

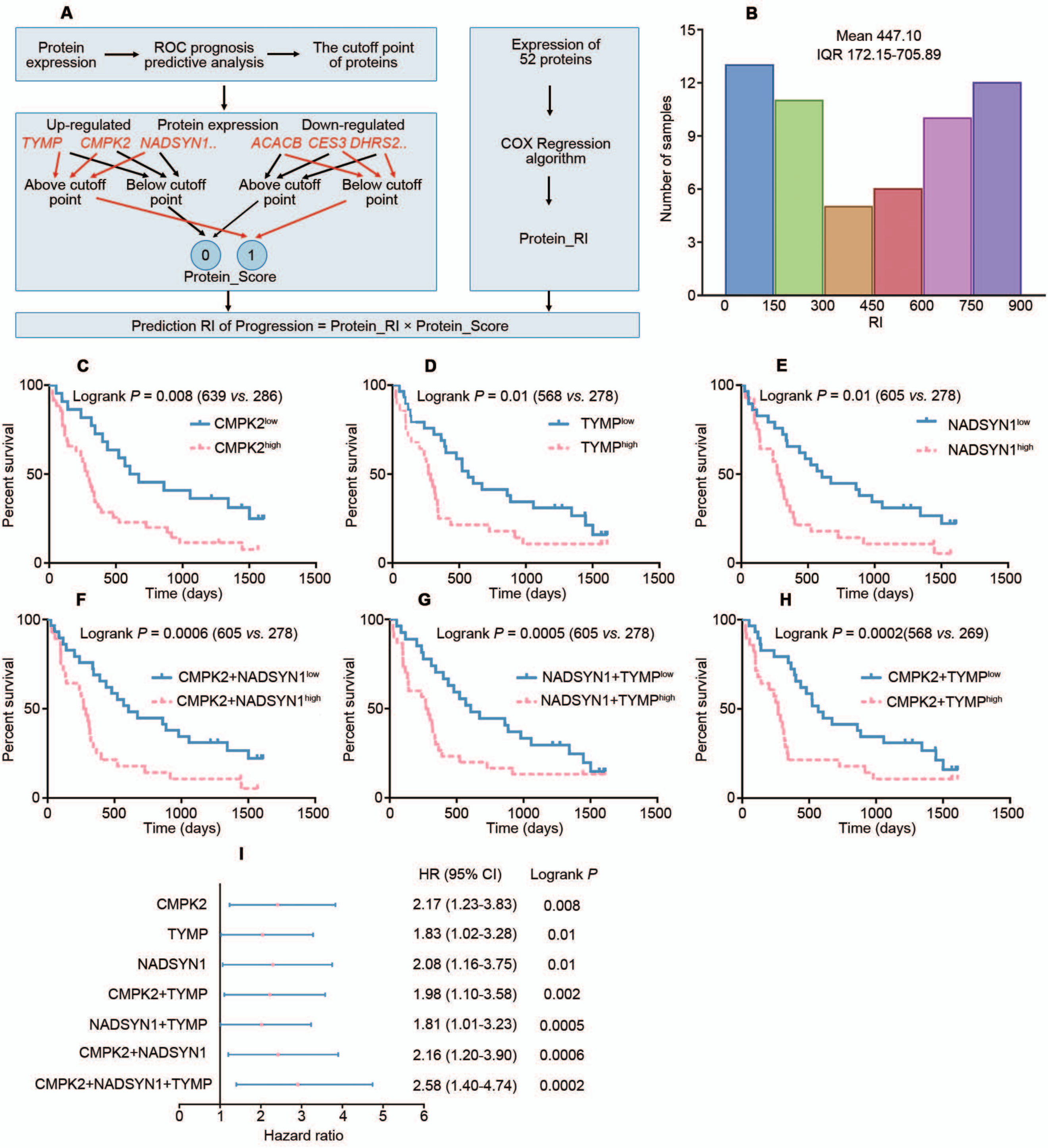

Fig. S4 Consensus matrix, CDF curve, and delta curve for all clusters.

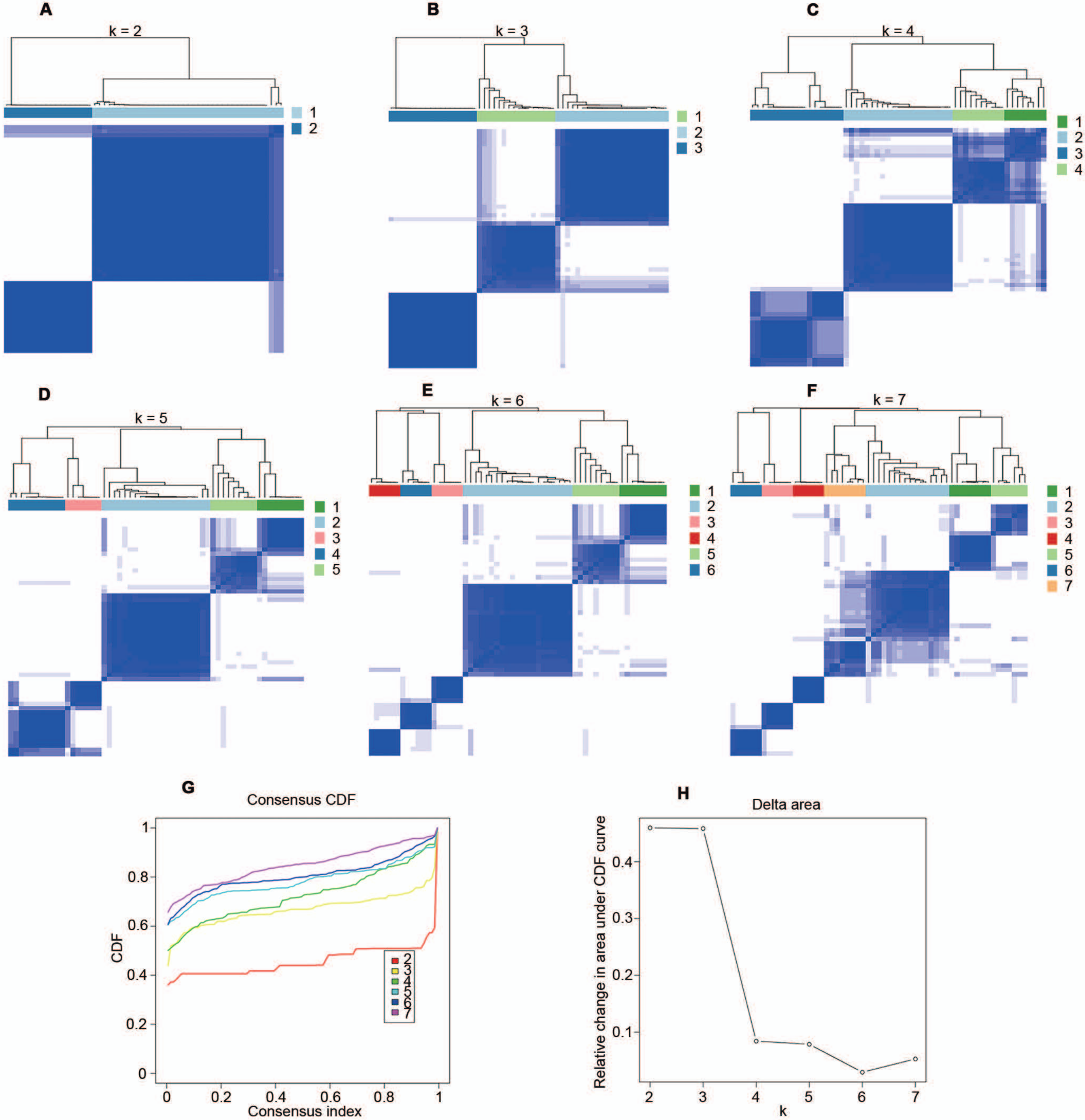

Fig. S5 Verification of TYMP expression related with progression of HCC and PECAM1 (CD31).

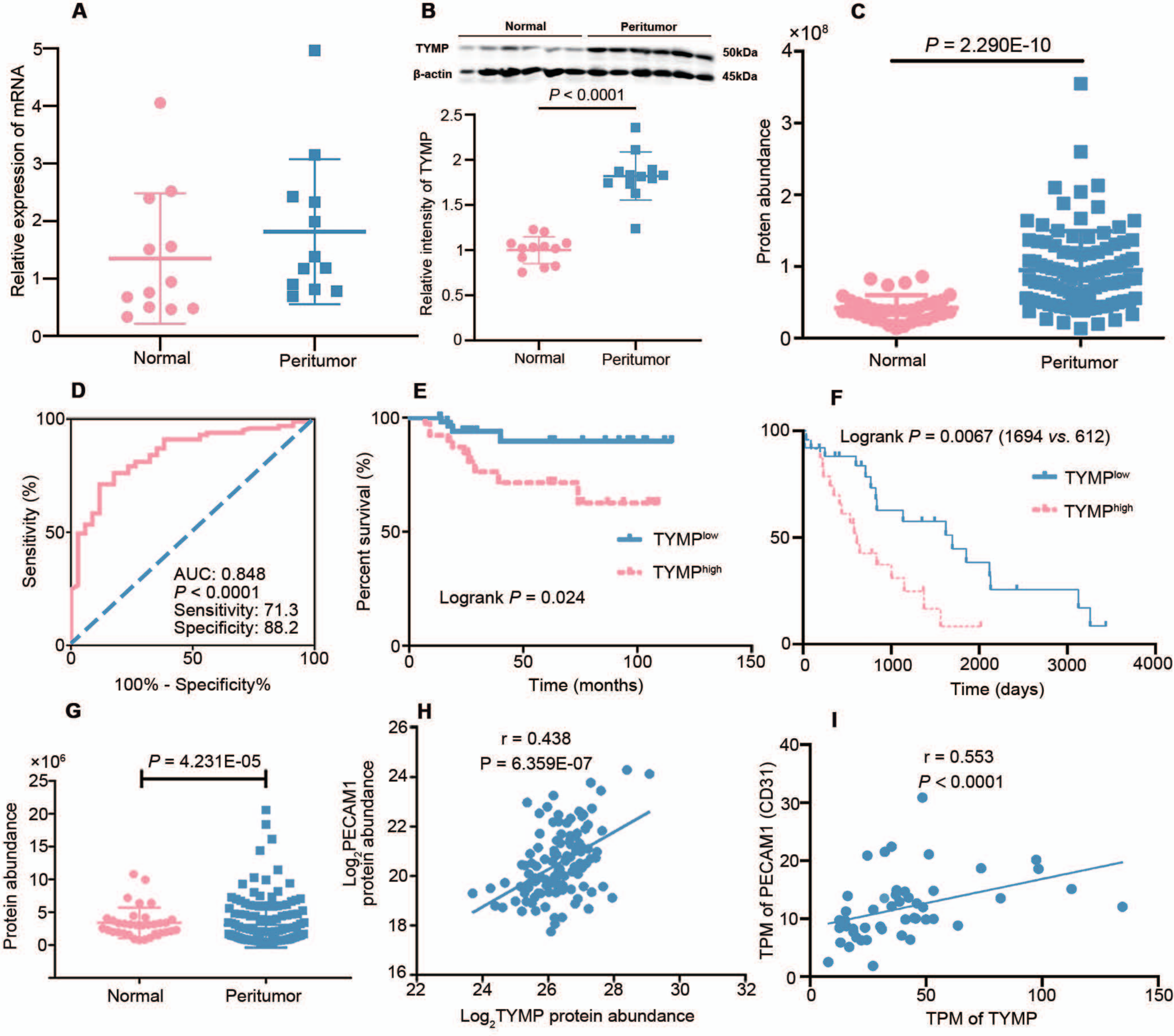

Fig. S6 TYMP expression related with signaling pathways in angiogenesis.

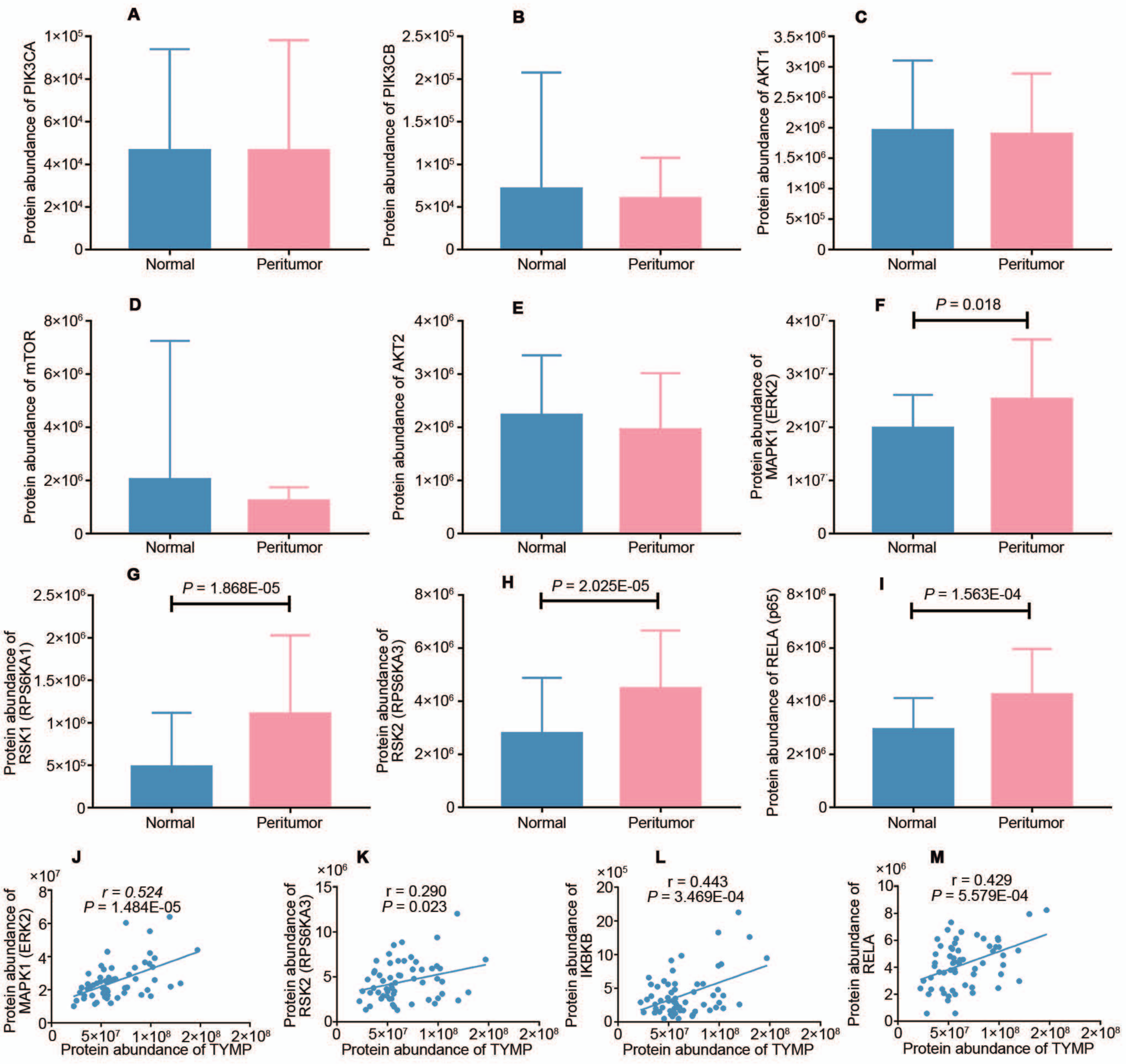

Fig. S1 Workflow of sample processing, quality control. (A) Workflow for quantitative proteomic analysis of human liver samples, including sample collection, preparation, liquid chromatography with tandem mass spectrometry (LC-MS/MS) detection and data processing. (B) Quality control for mass spectrometry. Distribution of the log_2-transformed protein abundance of identified proteins from a tryptic digest of HEK293T cells. In the box plots, the middle bar represents the median, and the box represents the interquartile range (IQR). (C) Longitudinal quality control of mass spectrometry using a tryptic digest of HEK293T cells. The top-right half of the panel represents the pairwise Spearman correlation coefficients of the samples, and the bottom-left half of the panel depicts pairwise scatter plots of the same comparison. (D) Screening of qualified human liver tissue samples. Number of proteins identified in 112 human liver tissue samples. Blue indicates normal liver tissue samples, and red indicates peritumor samples from HCC patients. The gray bars in the figure represent the unqualified sample of the protein identification number. (E) Distribution of the log_2-transformed iBAQ abundance of proteins identified in 95 proteome samples that passed quality control. Green represents normal samples (n = 34), and red denotes peritumor samples (n = 61). In the box plots, the middle bar represents the median, and the box represents the interquartile range; bars extend to 1.5x the IQR. (F) Subcellular distribution of total identified proteins annotated with Gene Ontology.

Fig. S2 Establishment of prediction scores and a prediction model of HCC occurrence. (A) Workflow for RIs calculations. Proteins related to HCC occurrence were divided into six categories. Logistic regression analysis was used to evaluate the contribution of each protein. The regression coefficient β from logistic regression analysis was considered as the RI for each

protein. Protein RIs of the same risk factor were added and then divided by the sum of the RIs for each patient to determine the RI of each risk factor. (B) Frequency distribution of the RI for HCC patients (n = 61). (C-E) ROC curves for HCC occurrence relative to expression of HSPA4L (C), VIL1 (D) and TYMP (E). (F-H) ROC curves for HCC occurrence relative to combined expression of HSPA4L and VIL1 (F), HSPA4L and TYMP (G), and VIL1 and TYMP (H). normal, n = 34; peritumor, n = 61. RI, risk index.

Fig. S3 Establishment of prediction scores and a prediction model of HCC progression. (A) Workflow for RIs calculations. The proteins related to HCC progression were divided into six categories, and Cox Risk regression analysis was used to evaluate the contribution by protein. The regression coefficient β from Cox Risk regression analysis was considered as the RI for each protein. Protein RIs of the same risk factor were added and then divided by the sum of the RIs for each patient to determine the RI of each risk factor. (B) Frequency distribution of RI for HCC patients (n = 57). (C-H) Kaplan-Meier curve analysis of survival relative to expression of CMPK2 (C), TYMP (D) and NADSYN1 (E), combined expression of CMPK2 and NADSYN1 (F), NADSYN1 and TYMP (G), and CMPK2 and TYMP (H). (I) Comparison of hazard ratios (HRs) for single or combined expression of CMPK2, TYMP and NADSYN1 in HCC patients (n = 57). RI, risk index.

Fig. S4 Consensus matrix, CDF curve, and delta curve for all clusters. (A-F) Consensus matrices for the 57 HCC samples from k = 2 to k = 7. Consensus clustering was performed for 6 947 proteins upon their abundance. (G) Cumulative distribution function (CDF) plots corresponding to consensus matrices from k = 2 to k = 7. (H) Delta plot assessing changes in the consensus CDF area used to determine the ideal number of clusters through a comparison

of k with k + 1. The three-cluster solution produced the largest k that resulted in the smallest incremental change in the consensus CDF area.

Fig. S5 Verification of TYMP expression related with progression of HCC and PECAM1 (CD31). (A) TYMP mRNA expression in normal (n =12) and peritumor (n =12) samples in our dataset. Data are presented as the mean ± SD. (B) Protein expression of TYMP in normal (n =12) and peritumor (n =12) samples in our dataset by western blot. Data are presented as the mean ± SD. (C) Quantification of TYMP levels in normal and peritumor samples in validation dataset. (D-E) Relationship between TYMP expression and the occurrence and progression of HCC in validation dataset. (F) Relationship between TYMP expression and progression of HCC in TCGA dataset. (G) Significant increase in PECAM1 expression in peritumor tissue in our validation dataset. (H-I) Positive correlation between expression of TYMP and PECAM1 in validation dataset (H) and TCGA dataset (I).

Fig. S6 TYMP expression related with signaling pathways in angiogenesis. (A-I) Changes in expression of proteins involved in angiogenesis-related signaling pathways in the PME of HCC patients. Expression of proteins related with PI3K/AKT/mTOR signaling pathway (A-E) and MAPK1 (ERK2)/RASK1/NF-κB signaling pathway (F-I). Data are presented as the mean ± SD. (J-M) Correlation of protein expression between TYMP and MAPK1 (J), TYMP and RSK2 (K), TYMP and IKBKB (L), TYMP and RELA (M).

Supplement methods

Liver tissue collection criteria

All tissues were provided by subjects of Han nationality who were 18 years-old or older. For normal tissues, most of donors were patients with hepatic haemangioma. All biochemical indicators of liver function, imaging examination and histopathology examination were normal. Patients who met the following criteria were excluded: (i) family history of inherited liver disease; (ii) hepatitis virus infection; alcoholic or nonalcoholic steatohepatitis, primary biliary cirrhosis, or autoimmune liver disease; (iii) bacterial and parasitic infections; (iv) other liver diseases (e.g., Budd-Chiari syndrome); (v) long-term use of drugs that could damage liver function or exposure to toxic substances; (vi) other diseases that affect liver function, such as heart disease, diabetes, renal insufficiency, connective tissue diseases, autoimmune diseases or Reye syndrome; and (vii) pregnancy. For peritumor tissues, the tissues adjacent to the tumor were approximately 2 cm away from the tumor and were taken from patients with HCC who did not receive tumor radiotherapy, tumor chemotherapy or targeted drug therapy before surgery. All liver tissues were washed with normal saline (precooled at 4 °C), weighed within 30 minutes, and stored in liquid nitrogen.

Collection of clinical information

Clinical information was recorded, including basic information (e.g., sociodemographic data, smoking and drinking status, medication history, family history, diagnosis) and information specific to the liver such as cirrhosis stage, maximum tumor diameter, presence of single or multiple tumors, and blood biochemical indicators (Table S1). The follow-up period began after surgery and lasted for 42~54 months. The survival time was determined

based on the time of death of the patient or the date of the last follow-up.

Proteomic processing of liver tissue

Liver protein extraction and digestion

Liver tissue was cut into 1mm^3 pieces, resuspended in phosphate-buffered saline (PBS) and centrifuged at 1000×g for 5 minutes at 4 °C. Then, 8 M urea (Sigma-Aldrich, Missouri, USA) containing protease inhibitor (Promega, Wisconsin, USA) at a 1:10 (W/V) ratio was added before sonication on ice (30 cycles of 3 s on and 3 s off). The lysate was centrifuged at 16 000×g for 20 minutes at 4 °C, and the supernatant was collected. The BCA method (Boster Biological Technology, Wuhan, China) was used to determine protein concentrations. Protein extracts were stored at -80 °C.

A filter-aided sample preparation method was used to digest the proteins. Lysate samples containing the equivalent of 250 μg protein were diluted into a final volume of 100 μL with 8 M urea buffer solution before 1 M DTT was added and vortexed for 1 minute. The samples were incubated at 37 °C for 4 h. After incubation, 1 M iodoacetamide (IAA) was added, and the mixture was vortexed for 1 minute. The samples were incubated at room temperature in the dark for 30 minutes. The solution was transferred to an ultracentrifugation tube and centrifuged at 14 000×g for 10 minutes. The ultracentrifugation tube was washed three times with 200 μL 8 M urea by centrifugation at 14 000×g for 15 minutes. Then, 200 μL 50 mM ammonium bicarbonate solution was used to wash the ultracentrifugation tube three times by centrifugation at 14 000×g for 15 minutes. Trypsin dissolved in 50 mM ammonium bicarbonate was added to the sample at a 50:1 ratio of protein:enzyme. The ultracentrifugation tube was placed in a 37 °C incubator for 16 h. The sample was then transferred to a fresh

ultracentrifugation tube that was centrifuged at 14 000×g for 15 minutes before addition of 100 μL 50 mM ammonium bicarbonate solution and centrifugation at 14 000×g for 15 minutes. The peptide solution was collected after protein digestion. A Nanodrop 2000c micro ultraviolet spectrophotometer (Thermo Fisher Scientific, Massachusetts, USA) was used to determine peptide concentrations. Peptides were recovered by heat-drying in a vacuum centrifuge at 45 °C and stored at -80 °C. The operator was blinded to the group of liver tissues.

Preparation of quality control peptide solution

To monitor the performance of mass spectrometry, a peptide solution produced from HEK293T cells was used as a quality control standard. HEK293T cells were treated according to the steps used for liver protein extraction and digestion. The detection process and mass spectrometer parameter settings used for the HEK293T cell peptide solution were completely consistent with those used for human liver tissue samples.

High-pH reverse-phase prefractionation of peptides

Peptide fractions were pre-separated using reverse chromatography columns. Briefly, a 3M Empore C8 solid phase extraction disk was placed on the bottom of a pipette tip, and 5 mg C18 medium was added. Acetonitrile and aqueous ammonia (pH 10.0) were added sequentially to equilibrate the tip. The dried peptides were dissolved in aqueous ammonia and transferred to the tip. Aqueous ammonia was added for desalination. Peptides were eluted with gradient proportions of acetonitrile and ammonia (6%, 9%, 12%, 15%, 18%, 21%, 25%, 30%, 35% and 50%). Then, the four gradient fractions of 25%, 30%, 35% and 50% were combined with those for 6%, 9%, 12% and 21%, respectively. Peptide fractions were

recovered in a vacuum centrifuge at 45 °C and stored at -80 °C.

Liquid chromatography tandem mass spectrometry analysis

The peptide fractions were dissolved in 0.1% formic acid (FA) aqueous solution and centrifuged at 14 000×g for 10 minutes. Peptide fractions were analyzed using liquid chromatography-mass spectrometry-tandem mass spectrometry (Q-Exactive HF LC-MS/MS).

Identification and quantification of protein by MaxQuant

Raw data from mass spectrometry were analyzed against the human UniProt protein sequence database (version 20140922, 20 193 sequences) with MaxQuant software (version 1.5.3.8). Trypsin was chosen as the digestion enzyme. The maximum number of enzyme-missing sites was 2. Cysteine urea methylation modification was set as the fixed modification. Methionine oxidation and protein N-terminal acetylation were set as variable modifications. The false discovery rate (FDR) for peptides was set to <1%. The maximum mass error of the primary ion and product ion scans for the initial mass spectrometry were set to 20 ppm and 0.5 Da, respectively. The corrected error of the precursor ion was set to 5 ppm. Peritumor tissues were grouped by median survival time, AFP content, cirrhosis stage, maximum tumor diameter and presence of single tumor or multiple tumors, and the number of identified proteins was then compared.

Normalization of proteomic data

A 6 947×95 protein expression matrix was obtained from the 3.2.4 MaxQuant result file. Intensity-based absolute protein quantification (iBAQ) based on peak intensity was used to express protein expression levels. The R/Bioconductor package limma v.3.24.15 was used to apply the normalized quantile function to normalize expression matrix quantiles. The $\log_2$

transformation of normalized iBAQ kurtosis was used for all quantitative analyses. The distribution of the proteome kurtosis among the samples was relatively balanced, and the distribution trend was consistent, indicating good consistency for the proteome quantification of 95 samples.

Proteomic characterization of the PME-O

Proteome for the PME-O

Receiver Characteristic Operator (ROC) curve analysis was used to analyze the differential proteome (n = 1 360) of the PME. All liver tissues were divided into two groups: PME and normal liver tissues. For each protein, $P < 0.05$ and an AUC ≥ 0.75 were regarded as the limits. To exclude irrelevant proteins, the exclusion criteria were formulated as: (i) no research related to tumors; (ii) function unknown; (iii) proteins related to liver fibrosis or liver cirrhosis; (iv) non-secreted proteins related to tumor cell proliferation, migration and invasion; (v) HBV-related proteins and no research related to tumors; and (vi) no direct or indirect relationship with known tumorigenesis factors. The remaining proteins were considered highly correlated with the PME occurrence proteome.

RI and prediction of HCC occurrence

Proteins related to HCC occurrence were divided into six categories: immunity, inflammation, angiogenesis, DNA damage and repair, metabolism and others. Logistic regression analysis was used to evaluate the contribution of each protein. The regression coefficient β from logistic regression analysis was considered as the RI for each protein. The cutoff point of the ROC curve analysis was taken as the boundary. A protein was scored as 1 point if the iBAQ was beyond the boundary, otherwise the assigned score was 0 points. Based on the formula

"Protein_RI" × "Protein_Score", the RIs of 40 proteins from 61 patients were calculated as the RIs of HCC occurrence, and a 40×61 protein RIs matrix were obtained (Fig. S3A).

According to the classification of proteins, protein RIs of the same risk factor were added and then divided by the sum of the RIs for each patient to determine the RI of each risk factor. To validate the clinical significance of the RI, the 40 protein RIs for each patient were summed as the patient RI, and the patient RIs were divided into two groups according to AFP content, cirrhosis stage and CYP2E1 activity. The relative fraction of each group was analyzed by a Chi-square test.

According to the AUC of ROC curve analysis and the weight of each protein, the immune protein HSPA4L, the inflammatory protein VIL1 and the angiogenesis protein TYMP were selected to establish a prediction model for HCC occurrence. Binary logistic regression was used to calculate the probability value of prediction, and then the value was used to calculate the AUC of ROC curve analysis. The prediction model equation of HCC occurrence is Logit (P) = 2.190 + 3.869 × HSPA4L - 1.285 × VIL1 + 1.631 × TYMP, and probability of HCC occurrence risk is $P = 1 / [1 + e^{-(2.190 + 3.869 \times \text{HSPA4L} - 1.285 \times \text{VILI} + 1.631 \times \text{TYMP})]}$. To verify the HCC occurrence prediction model, proteomics data from the PRoteomics IDEntification (PRIDE) database (www.ebi.ac.uk/pride/archive, accession numbers PXD006512) were downloaded for verification. The validation dataset PXD006512 was tested in the same methods (label-free) and institution (State Key Laboratory of Proteomics, Beijing, China) with our data. It is available, which is comparable and suitable for verification. The iBAQ values of HSPA4L, VIL1, and TYMP in the verification data were calculated for each patient using a prediction model. Based on the prediction probability value of the verification data, ROC curve analysis

was used to evaluate the prediction model.

Proteomic characterization of the PME-P

Proteome of the PME-P

Kaplan-Meier survival curve analysis was performed on the differential proteome (n = 1 360) in the PME, and $P < 0.05$ was considered the limit. Then, the remaining proteins were analyzed by ROC curve analysis. All PME samples were divided into two groups according to the median of iBAQ value of each protein with an AUC ≥ 0.7 and $P < 0.05$ as limiting conditions. Based on the literature, a high correlation with the HCC progression proteome was obtained. The exclusion criteria were the same as those for occurrence except: (vii) no direct or indirect relationship with known tumor progression factors.

RI and prediction of HCC progression

The proteins related to HCC progression were divided into six categories, and logistic regression analysis was used to evaluate the contribution by protein. The method was similar with that for HCC occurrence (Fig. S3a). The Spearman rank correlation test was used to analyze the correlation between the prediction RI of HCC progression and survival time of patients.

According to the AUC of ROC curve analysis, the top three proteins CMPK2, TYMP and NADSYN1 were selected to establish a prediction model for HCC progression. The iBAQ values of the proteins CMPK2, TYMP and NADSYN1 were $\log_2$-transformed, and the prognostic risk function equation was obtained according to Cox Risk Regression analysis. The equation of prognostic risk is $h(t) = h_0(t) \exp(0.382 \times \text{CMPK2} + 0.10 \times \text{TYMP} + 0.324 \times \text{NADSYN1})$ and the equation of prognostic index is PI = 0.382 × CMPK2 + 0.10 × TYMP

+ 0.324 × NADSYN1. The HR was used to evaluate the advantages of the combined three-protein prediction model. Proteomics data from the PRIDE database (www.ebi.ac.uk/pride/archive, accession number PXD006512) were downloaded for verification.

Comparison of risk factors for the occurrence and progression of HCC

ROC curve analysis was used to analyze the proteins for occurrence and progression. All liver tissues were divided into two groups: PME and normal liver tissues and the number of proteins was counted with an AUC ≥ 0.7 as boundary. Kaplan-Meier survival curve analysis was performed on the occurrence and progression proteins. All PME samples were divided into two groups according to the median of iBAQ value of each protein. The number of proteins was counted with P <0.05 as boundary. Chi-square test and Fisher Exact tests were used to show the difference of occurrence and progression proteins in predicting occurrence and progression.

Liver orthotopic transplantation tumor model with H22 cell lines in mice

Male BALB/c mice (n = 47, 6~8 weeks) were purchased from Vital River Laboratory Animal Technology Co., Ltd. (Beijing, China) and divided into 4 groups: the sham operation group (n = 10), model group (n = 13), TPI group (n = 12) and bevacizumab group (n = 12). In the experiment of Kin59, male BALB/c mice (n = 33, 6~8 weeks) were divided into 3 groups: the sham operation group (n = 9), model group (n = 12) and Kin59 group (n = 12). The mice were housed in a room with controlled humidity (50~70%) and temperature (18~22 °C) with free access to food and water. After adaptive feeding for one week, the mice were transplanted with HCC cells.

H22 cells were purchase from China Center for Type Culture Collection (Wuhan, China). H22 cells in the logarithmic growth phase were centrifuged at 1 000 rpm/min for 5 minutes and the supernatant was discarded. The cells were resuspended in sterile saline and were counted by microscopy. The cell concentration was adjusted to 1.5×10^6/mL. BALB/c mice were anesthetized by intraperitoneal injection of 5% chloral hydrate, fixed in the supine position on a field disinfected with iodophor, and a longitudinal incision was made from under the xiphoid process along the abdominal midline to fully expose the left lobe of the liver. A suspension of H22 cells (10 μL) was slowly injected with a 25 μL microsyringe (10 μL sterile saline was injected for the sham operation group). The operator was blinded to the group of mice. After the injection, the needle was temporarily kept in place and sterile gauze was used to gently press the hole left by the needle until bleeding from the liver stopped. The abdomen was then sutured layer by layer.

The intervention group was given 100 mg/kg tipiracil 3 days before the operation and once a day until the end of the experiment; the bevacizumab group was given 5 mg/kg bevacizumab twice a week until the end of the experiment; the sham operation group and the model group were given an equal volume of normal saline. After 21 days, the mice were sacrificed; the livers were collected, weighed, and photographed; and tumors were measured to calculate the tumor growth inhibition rate. In the experiment of KIN59, the intervention group was given 30 mg/kg kin59 (in 20% DMSO, 20% cremophore in PBS) 3 days before the operation. Other processes are the same as above. All operations were approved by the Medical Ethics Committee of Zhengzhou University.

Tumor growth inhibition rate = [(Wm -Wg)/Wm] ×100%

Wm: average tumor weight in the model group; Wg: average tumor weight in the intervention group.
