# Supplementary material for: The proteomic characterization of the peritumor microenvironment in human hepatocellular carcinoma
Source: Oncogene. 2022 Mar 21;41(17):2480–91. doi: 10.1038/s41388-022-02264-3 (PMC9033583; doi:10.1038/s41388-022-02264-3)
Supplement: Supplementary file 2 — Supplement Tables [file 41388_2022_2264_MOESM2_ESM.docx]

**Supplement Table**

**Table S1 Demographic data and clinical characteristics of liver tissue samples from normal and HCC patients**

| Characteristic | Normal (N=41) | HCC (N=71) | *P* value |
| --- | --- | --- | --- |
| Age (years) |  |  |  |
| ≤55, n (%) | 33 (80.5) | 37 (52.1) | **0.003** |
| >55, n (%) | 8 (19.5) | 34 (47.9) |  |
| Gender |  |  |  |
| Male, n (%) | 20 (48.8) | 59 (83.1) | **1.241E-04** |
| Female, n (%) | 21 (51.2) | 12 (16.9) |  |
| Smoking status |  |  |  |
| Present, n (%) | 10 (24.4) | 33 (46.5) | **0.021** |
| Absent, n (%) | 31 (75.6) | 38 (53.5) |  |
| Drinking status |  |  |  |
| Present, n (%) | 8 (19.5) | 27 (38.0) | **0.042** |
| Absent, n (%) | 33 (80.5) | 44 (62.0) |  |
| AFP (ng/ml) | 2.22 (1.16-2.76) | 304.8 (8.22-1210) | **8.031E-09** |
| ALT (U/L) | 19 (13~27) | 40 (26~58) | **1.530E-05** |
| AST (U/L) | 19 (15~25) | 40 (30~59) | **1.658E-08** |
| ALP(U/L) | 72 (62-88) | 102 (80-132) | **5.346E-05** |
| GGT (U/L) | 24 (16~47) | 75 (48~118) | **1.175E-05** |
| TP (g/L) | 66.5 (61.6~73.1) | 70.3 (67.2~73.7) | **0.036** |
| TBIL (μmol/L) | 7.5 (5.9~11.2) | 13.0 (9.2~19.3) | **3.253E-06** |
| PT (second) | 11.6 (10.7-12.2) | 12.6 (12.0-13.7) | **7.947E-07** |
| Diagnosis | Liver hemangioma,  cholelithiasis | HCC |  |
| Pathology classification | Normal | S1, S2, S3, S4 |  |
| Cirrhosis stage |  |  |  |
| ≤2，n (%) |  | 17 (25.4) |  |
| >2, n (%) |  | 50 (74.6) |  |
| Max diameter (cm) |  |  |  |
| ≤8, n (%) |  | 42 (64.6) |  |
| >8, n (%) |  | 23 (35.4) |  |
| Tumor number |  |  |  |
| Single, n (%) |  | 40 (61.5) |  |
| Mutiple, n (%) |  | 25 (38.5) |  |
| CYP2E1 activity |  |  |  |
| Negative, n (%) | 29 (96.7) | 31 (53.4) | **3.691E-05** |
| Positive, n (%) | 1 (3.3) | 27 (46.6) |  |

**Table S2 Functional classification of proteins for prediction of HCC occurrence**

| **Category** | **Gene** | **Protein_ID** | **Protein** |
| --- | --- | --- | --- |
| **Immunity** | HSPA4L | O95757 | Heat shock 70 kDa protein 4L |
|  | CD74 | P04233 | HLA class II histocompatibility antigen  gamma chain |
|  | C1QB | P02746 | Complement C1q subcomponent subunit B |
|  | CLEC4G | Q6UXB4 | C-type lectin domain family 4 member G |
|  | FGL2 | Q14314 | Fibroleukin (Fibrinogen-like protein 2) |
|  | PSMB8 | P28062 | Proteasome subunit beta type-8 |
|  | TAP2 | Q03519 | Antigen peptide transporter 2 |
|  | TAPBP | O15533 | Tapasin (TAP-binding protein) |
|  | TSPAN33 | Q86UF1 | Tetraspanin-33 |
|  | CXCL12 | P48061 | Stromal cell-derived factor 1;  SDF-1-beta (3-72) |
| **Inflammation** | VIL1 | P09321 | Villin-1 |
|  | A2M | P01023 | Alpha-2-macroglobulin |
|  | APCS | P02743 | Serum amyloid P-component |
|  | LGALS4 | P56470 | Galectin-4 |
|  | PLCG2 | P16885 | 1-phosphatidylinositol 4,5-bisphosphate  phosphodiesterase gamma-2 |
|  | PYCARD | Q9ULZ3 | Apoptosis-associated speck-like  protein containing a CARD |
|  | STAT1 | P42224 | Signal transducer and activator  of transcription 1-alpha/beta |
| **Angiogenesis** | TYMP | P19971 | Thymidine phosphorylase |
|  | ANGPTL6 | Q8NI99 | Angiopoietin-related protein 6 |
|  | EFEMP1 | Q12805 | EGF-containing fibulin-like  extracellular matrix protein 1 |
|  | FBLN5 | Q9UBX5 | Fibulin-5 |
|  | MFAP4 | P55083 | Microfibril-associated glycoprotein 4 |
|  | MFI2 | P08582 | Melanotransferrin |
|  | NPNT | Q6UXI9 | Nephronectin |
|  | ACE | P12821 | Angiotensin-converting enzyme; |
| **Metabolism** | CYP51A1 | Q16850 | Lanosterol 14-alpha demethylase |
|  | GALE | Q14376 | UDP-glucose 4-epimerase |
|  | GALK1 | P51570 | Galactokinase |
|  | OXCT1 | P55809 | Succinyl-CoA:3-ketoacid coenzyme A  transferase 1 |
| Proliferation and invasion | INHBE | P58166 | Inhibin beta E chain |
|  | LOXL1 | Q08397 | Lysyl oxidase homolog 1 |
|  | EFEMP2 | O95967 | EGF-containing fibulin-like  Extracellular matrix protein 2 |
|  | LTBP1 | Q14766 | Latent-transforming growth factor  beta-binding protein 1 |
|  | LTBP4 | Q8N2S1 | Latent-transforming growth factor  beta-binding protein 4 |
|  | TGFBI | Q15582 | Transforminggrowth factor-beta-  induced protein ig-h3 |
|  | LGALS3BP | Q08380 | Galectin-3-binding protein |
| DNA damage and repair | CYP3A7 | P24462 | Cytochrome P450 3A7 |
|  | ABCB10 | Q9NRK6 | ATP-binding cassette sub-family  B member 10 |
|  | GPX2 | P18283 | Glutathione peroxidase 2 |
|  | GNMT | Q14749 | Glycine N-methyltransferase |

**Table S3 Prediction RI of 40 proteins for the PME-O**

| **Protein** | **Risk_Index** | **Protein_expression** | **Protein_Score** |
| --- | --- | --- | --- |
| **CD74** | **14.9816** | **Above cutoff point** | **1** |
|  |  | **Below cutoff point** | **0** |
| **TYMP** | **13.4278** | **Above cutoff point** | **1** |
|  |  | **Below cutoff point** | **0** |
| **A2M** | **11.5790** | **Above cutoff point** | **1** |
|  |  | **Below cutoff point** | **0** |
| **PYCARD** | **8.0288** | **Above cutoff point** | **1** |
|  |  | **Below cutoff point** | **0** |
| **STAT1** | **7.9518** | **Above cutoff point** | **1** |
|  |  | **Below cutoff point** | **0** |
| **TAPBP** | **7.8005** | **Above cutoff point** | **1** |
|  |  | **Below cutoff point** | **0** |
| **LTBP1** | **7.5464** | **Above cutoff point** | **1** |
|  |  | **Below cutoff point** | **0** |
| **LOXL1** | **7.2441** | **Above cutoff point** | **1** |
|  |  | **Below cutoff point** | **0** |
| **HSPA4L** | **6.1551** | **Above cutoff point** | **1** |
|  |  | **Below cutoff point** | **0** |
| **GALK1** | **5.8177** | **Above cutoff point** | **0** |
|  |  | **Below cutoff point** | **1** |
| **MFAP4** | **5.6371** | **Above cutoff point** | **1** |
|  |  | **Below cutoff point** | **0** |
| **FGL2** | **4.7886** | **Above cutoff point** | **1** |
|  |  | **Below cutoff point** | **0** |
| **TAP2** | **4.7635** | **Above cutoff point** | **1** |
|  |  | **Below cutoff point** | **0** |
| **CLEC4G** | **4.3679** | **Above cutoff point** | **1** |
|  |  | **Below cutoff point** | **0** |
| **EFEMP1** | **4.2890** | **Above cutoff point** | **1** |
|  |  | **Below cutoff point** | **0** |
| **ABCB10** | **3.9483** | **Above cutoff point** | **1** |
|  |  | **Below cutoff point** | **0** |
| **VIL1** | **3.8030** | **Above cutoff point** | **1** |
|  |  | **Below cutoff point** | **0** |
| **GPX2** | **3.7002** | **Above cutoff point** | **1** |
|  |  | **Below cutoff point** | **0** |
| **PSMB8** | **3.6540** | **Above cutoff point** | **1** |
|  |  | **Below cutoff point** | **0** |
| **NPNT** | **3.4757** | **Above cutoff point** | **1** |
|  |  | **Below cutoff point** | **0** |
| **PLCG2** | **3.3160** | **Above cutoff point** | **1** |
|  |  | **Below cutoff point** | **0** |
| **LTBP4** | **3.2293** | **Above cutoff point** | **1** |
|  |  | **Below cutoff point** | **0** |
| **C1QB** | **3.2013** | **Above cutoff point** | **1** |
|  |  | **Below cutoff point** | **0** |
| **MFI2** | **2.7484** | **Above cutoff point** | **1** |
|  |  | **Below cutoff point** | **0** |
| **INHBE** | **2.7351** | **Above cutoff point** | **1** |
|  |  | **Below cutoff point** | **0** |
| **GNMT** | **2.5773** | **Above cutoff point** | **1** |
|  |  | **Below cutoff point** | **0** |
| **EFEMP2** | **2.5645** | **Above cutoff point** | **1** |
|  |  | **Below cutoff point** | **0** |
| **APCS** | **2.5185** | **Above cutoff point** | **1** |
|  |  | **Below cutoff point** | **0** |
| **FBLN5** | **2.3220** | **Above cutoff point** | **1** |
|  |  | **Below cutoff point** | **0** |
| **CYP3A7** | **2.2715** | **Above cutoff point** | **1** |
|  |  | **Below cutoff point** | **0** |
| **ACE** | **1.9703** | **Above cutoff point** | **1** |
|  |  | **Below cutoff point** | **0** |
| **CYP51A1** | **1.9400** | **Above cutoff point** | **1** |
|  |  | **Below cutoff point** | **0** |
| **LGALS3BP** | **1.7153** | **Above cutoff point** | **1** |
|  |  | **Below cutoff point** | **0** |
| **TGFBI** | **1.4226** | **Above cutoff point** | **1** |
|  |  | **Below cutoff point** | **0** |
| **TSPAN33** | **1.1632** | **Above cutoff point** | **1** |
|  |  | **Below cutoff point** | **0** |
| **OXCT1** | **0.8043** | **Above cutoff point** | **1** |
|  |  | **Below cutoff point** | **0** |
| **GALE** | **0.6891** | **Above cutoff point** | **1** |
|  |  | **Below cutoff point** | **0** |
| **ANGPTL6** | **0.5580** | **Above cutoff point** | **1** |
|  |  | **Below cutoff point** | **0** |
| **CXCL12** | **0.4807** | **Above cutoff point** | **1** |
|  |  | **Below cutoff point** | **0** |
| **LGALS4** | **0.3887** | **Above cutoff point** | **1** |
|  |  | **Below cutoff point** | **0** |

**Table S4 Functional classification of proteins for prediction of HCC progression**

| **Category** | **Gene** | **Protein_ID** | **Protein** |
| --- | --- | --- | --- |
| Immunity | ARPC1B | O15143 | Actin-related protein 2/3 complex  subunit 1B |
|  | C1QC | P02747 | Complement C1q subcomponent  subunit C |
|  | C7 | P10643 | Complement component C7 |
|  | IGHG1 | P01857 | Ig gamma-1 chain C region |
|  | LGALS9 | O00182 | Galectin-9 |
|  | MZB1 | Q8WU39 | Marginal zone B and B1cell  specific protein |
|  | PSMB9 | P28065 | Proteasome subunit beta type-9 |
|  | PTMS | P20962 | Parathymosin |
| Inflammation | AKR1B1 | P15121 | Aldo-keto reductase family 1  member B1 |
|  | CASP1 | P29466 | Caspase-1 |
|  | CMPK2 | Q5EBM0 | Cytidine/Uridine Monophosphate  Kinase 2 |
|  | FAM213A | Q9BRX8 | Redox-regulatory protein  FAM213A |
|  | MVP | Q14764 | Major vault protein |
|  | MYL12A/ B | P19105 | Myosin regulatory light chain  12A/B |
|  | PTGIS | Q16647 | Prostacyclin synthase |
|  | S100A11 | P31949 | Protein S100-A11 |
|  | STK4 | Q13043 | Serine/threonine-protein kinase 4 |
|  | SUMF2 | Q8NBJ7 | Sulfatase-modifying factor 2 |
| Angiogenesis | ITGAV | P06756 | Integrin alpha-V |
|  | MYOF | Q9NZM1 | Myoferlin |
|  | NRP1 | O14786 | Neuropilin-1 |
|  | PECAM1 | P16284 | Platelet endothelial cell  adhesion molecule |
|  | PFN1 | P07737 | Profilin-1 |
|  | RNF213 | Q63HN8 | E3 ubiquitin-protein ligase  RNF213 |
|  | TAGLN2 | P37802 | Transgelin-2 |
|  | TYMP | P19971 | Thymidine phosphorylase |
|  | VCAN | P13611 | Versican core protein |
|  | VWF | P04275 | von Willebrand factor |
| Metabolism | ACACB | O00763 | Acetyl-CoA carboxylase 2 |
|  | BCAT2 | O15382 | Branched chain amino  acid aminotransferase |
|  | CES3 | Q6UWW8 | Carboxylesterase 3 |
|  | DHRS2 | Q13268 | Dehydrogenase/reductase SDR  family member 2 |
|  | FABP5 | Q01469 | Fatty acid-binding protein, epidermal |
|  | GAPDH | P04406 | Glyceraldehyde-3-phosphate dehydrogenase |
|  | GNPDA1 | P46926 | Glucosamine-6-phosphate isomerase 1 |
|  | GSTP1 | P09211 | Glutathione S-transferase P |
|  | GYS2 | P54840 | Glycogen synthase |
|  | NADSYN1 | Q6IA69 | Glutamine-dependent NAD(+)  synthetase |
|  | PDP1 | Q9P0J1 | [Pyruvate dehydrogenase  [acetyl-transferring]]-phosphatase 1 |
|  | PFKP | Q01813 | ATP-dependent 6-phosphofructokinase |
|  | RBP4 | P02753 | Retinol-binding protein 4 |
|  | SLC25A20 | O43772 | Mitochondrial carnitine/acylcarnitine  carrier protein |
|  | SLC27A5 | Q9Y2P5 | Bile acyl-CoA synthetase |
| DNAdamage  and repair | FAM129B | Q96TA1 | Niban-like protein 1 |
|  | GPX3 | P22352 | Glutathione peroxidase 3 |
|  | VCP | P55072 | Transitional endoplasmic  reticulum ATPase |
| Proliferation and invasion | ASPN | Q9BXN1 | Asporin |
|  | COL3A1 | P02461 | Collagen alpha-1(III) chain |
|  | IGFBP7 | Q16270 | Insulin-like growth factor-binding  protein 7 |
|  | LGALS3BP | Q08380 | Galectin-3-binding protein |
|  | TGFBI | Q15582 | Transforming growth factor-beta-  induced protein ig-h3 |
|  | TINAGL1 | Q9GZM7 | Tubulointerstitial nephritis antigen-like |

**Table S5 Prediction RI of 52 proteins for the PME-P**

| **Protein** | **Risk_Index** | **Protein_Expression** | **Protein_Score** |
| --- | --- | --- | --- |
| **SUMF2** | **65.8316** | **Above cutoff point** | **1** |
|  |  | **Below cutoff point** | **0** |
| **TYMP** | **48.9242** | **Above cutoff point** | **1** |
|  |  | **Below cutoff point** | **0** |
| **AKR1B1** | **47.6480** | **Above cutoff point** | **1** |
|  |  | **Below cutoff point** | **0** |
| **RNF213** | **45.4674** | **Above cutoff point** | **1** |
|  |  | **Below cutoff point** | **0** |
| **MVP** | **43.3127** | **Above cutoff point** | **1** |
|  |  | **Below cutoff point** | **0** |
| **FAM213A** | **42.9602** | **Above cutoff point** | **0** |
|  |  | **Below cutoff point** | **1** |
| **PFN1** | **42.5952** | **Above cutoff point** | **1** |
|  |  | **Below cutoff point** | **0** |
| **C7** | **40.1546** | **Above cutoff point** | **1** |
|  |  | **Below cutoff point** | **0** |
| **RBP4** | **33.7125** | **Above cutoff point** | **1** |
|  |  | **Below cutoff point** | **0** |
| **TGFBI** | **31.8865** | **Above cutoff point** | **1** |
|  |  | **Below cutoff point** | **0** |
| **ARPC1B** | **29.9239** | **Above cutoff point** | **1** |
|  |  | **Below cutoff point** | **0** |
| **LGALS3BP** | **28.6078** | **Above cutoff point** | **1** |
|  |  | **Below cutoff point** | **0** |
| **LGALS9** | **25.7911** | **Above cutoff point** | **1** |
|  |  | **Below cutoff point** | **0** |
| **GNPDA1** | **23.9446** | **Above cutoff point** | **1** |
|  |  | **Below cutoff point** | **0** |
| **VCP** | **23.7186** | **Above cutoff point** | **1** |
|  |  | **Below cutoff point** | **0** |
| **IGHG1** | **20.6397** | **Above cutoff point** | **1** |
|  |  | **Below cutoff point** | **0** |
| **MYL12A;** | **19.8024** | **Above cutoff point** | **1** |
| **MYL12B** |  | **Below cutoff point** | **0** |
| **GAPDH** | **18.8592** | **Above cutoff point** | **1** |
|  |  | **Below cutoff point** | **0** |
| **GSTP1** | **18.5594** | **Above cutoff point** | **1** |
|  |  | **Below cutoff point** | **0** |
| **C1QC** | **17.9941** | **Above cutoff point** | **1** |
|  |  | **Below cutoff point** | **0** |
| **ITGAV** | **17.7539** | **Above cutoff point** | **1** |
|  |  | **Below cutoff point** | **0** |
| **IGFBP7** | **17.2963** | **Above cutoff point** | **1** |
|  |  | **Below cutoff point** | **0** |
| **NADSYN1** | **17.0255** | **Above cutoff point** | **1** |
|  |  | **Below cutoff point** | **0** |
| **SLC27A5** | **16.8711** | **Above cutoff point** | **0** |
|  |  | **Below cutoff point** | **1** |
| **SLC25A20** | **16.1767** | **Above cutoff point** | **0** |
|  |  | **Below cutoff point** | **1** |
| **VWF** | **16.0179** | **Above cutoff point** | **1** |
|  |  | **Below cutoff point** | **0** |
| **PTMS** | **15.9168** | **Above cutoff point** | **1** |
|  |  | **Below cutoff point** | **0** |
| **PFKP** | **15.4569** | **Above cutoff point** | **1** |
|  |  | **Below cutoff point** | **0** |
| **BCAT2** | **14.7494** | **Above cutoff point** | **1** |
|  |  | **Below cutoff point** | **0** |
| **PECAM1** | **13.1180** | **Above cutoff point** | **1** |
|  |  | **Below cutoff point** | **0** |
| **S100A11** | **12.2259** | **Above cutoff point** | **1** |
|  |  | **Below cutoff point** | **0** |
| **GPX3** | **11.3056** | **Above cutoff point** | **1** |
|  |  | **Below cutoff point** | **0** |
| **MYOF** | **10.3285** | **Above cutoff point** | **1** |
|  |  | **Below cutoff point** | **0** |
| **VCAN** | **9.3562** | **Above cutoff point** | **1** |
|  |  | **Below cutoff point** | **0** |
| **NRP1** | **8.6977** | **Above cutoff point** | **1** |
|  |  | **Below cutoff point** | **0** |
| **FABP5** | **7.7308** | **Above cutoff point** | **1** |
|  |  | **Below cutoff point** | **0** |
| **TINAGL1** | **7.4972** | **Above cutoff point** | **1** |
|  |  | **Below cutoff point** | **0** |
| **COL3A1** | **7.1835** | **Above cutoff point** | **1** |
|  |  | **Below cutoff point** | **0** |
| **GYS2** | **6.1536** | **Above cutoff point** | **1** |
|  |  | **Below cutoff point** | **0** |
| **FAM129B** | **3.5995** | **Above cutoff point** | **1** |
|  |  | **Below cutoff point** | **0** |
| **DHRS2** | **3.2503** | **Above cutoff point** | **0** |
|  |  | **Below cutoff point** | **1** |
| **MZB1** | **3.1389** | **Above cutoff point** | **1** |
|  |  | **Below cutoff point** | **0** |
| **CASP1** | **2.9691** | **Above cutoff point** | **1** |
|  |  | **Below cutoff point** | **0** |
| **PTGIS** | **2.6049** | **Above cutoff point** | **1** |
|  |  | **Below cutoff point** | **0** |
| **CES3** | **2.6008** | **Above cutoff point** | **0** |
|  |  | **Below cutoff point** | **1** |
| **TAGLN2** | **1.3954** | **Above cutoff point** | **1** |
|  |  | **Below cutoff point** | **0** |
| **PDP1** | **1.1930** | **Above cutoff point** | **1** |
|  |  | **Below cutoff point** | **0** |
| **PSMB9** | **1.0221** | **Above cutoff point** | **1** |
|  |  | **Below cutoff point** | **0** |
| **ASPN** | **0.8483** | **Above cutoff point** | **1** |
|  |  | **Below cutoff point** | **0** |
| **ACACB** | **0.4337** | **Above cutoff point** | **0** |
|  |  | **Below cutoff point** | **1** |
| **STK4** | **0.2565** | **Above cutoff point** | **1** |
|  |  | **Below cutoff point** | **0** |
| **CMPK2** | **0.1084** | **Above cutoff point** | **1** |
|  |  | **Below cutoff point** | **0** |

**Table S6 Relationship between** **molecular subtypes and** **clinical characteristic**

| **Characteristic** | **S-Ⅰ (n=16)** | **S-Ⅱ (n=23)** | **S-Ⅲ (n=18)** | ***P* value** |
| --- | --- | --- | --- | --- |
| Age (years) |  |  |  |  |
| ≤55, n (%) | 6 (37.5) | 11 (47.8) | 9 (50) | 0.737 |
| >55, n (%) | 10 (62.5) | 12 (52.2) | 9 (50) |  |
| Gender |  |  |  |  |
| Male, n (%) | 13 (81.2) | 20 (87.0) | 16 (88.9) | 0.802 |
| Female, n (%) | 3 (18.8) | 3 (13.0) | 2 (11.1) |  |
| Smoking status |  |  |  |  |
| Present, n (%) | 10 (62.5) | 10 (43.5) | 11 (61.1) | 0.922 |
| Absent, n (%) | 6 (37.5) | 13 (56.5) | 7 (38.9) |  |
| Drinking status |  |  |  |  |
| Present, n (%) | 10 (62.5) | 16 (69.6) | 13 (72.2) | 0.821 |
| Absent, n (%) | 6 (37.5) | 7 (30.4) | 5 (27.8) |  |
| AFP (ng/ml) |  |  |  |  |
| ≤300 ng/ml, n (%) | 7 (50.0) | 10 (45.5) | 9 (56.2) | 0.806 |
| >300 ng/ml, n (%) | 7 (50.0) | 12 (54.5) | 7 (43.8) |  |
| Cirrhosis stage |  |  |  |  |
| ≤2，n (%) | 4 (25.0) | 5 (21.7) | 6 (33.3) | 0.698 |
| >2, n (%) | 12 (75.0) | 18 (78.3) | 12 (66.7) |  |
| Max diameter (cm) |  |  |  |  |
| ≤8, n (%) | 11 (73.3) | 16 (69.6) | 9 (52.9) | 0.415 |
| >8, n (%) | 4 (26.7) | 7 (30.4) | 8 (47.1) |  |
| Tumor number |  |  |  |  |
| Single, n (%) | 6 (40.0) | 15 (65.2) | 11(64.7) | 0.246 |
| Mutiple, n (%) | 9 (60.0) | 8 (34.8) | 6 (35.3) |  |
| CYP2E1 activity |  |  |  |  |
| Negative, n (%) | 11 (68.8) | 16 (69.6) | 6 (33.3) | **0.039** |
| Positive, n (%) | 5 (31.2) | 7 (30.4) | 12 (66.7) |  |
| Survival time |  |  |  |  |
| ≤median, n (%) | 7 (46.7) | 15 (75.0) | 12 (92.3) | **0.026** |
| >median, n (%) | 8 (53.3) | 5 (25.0) | 6 (7.7) |  |

**Table S7 Potential drug targets for the PME of HCC**

|  | **Gene** | **Protein** | **Function** | **Risk**  **index** | **Drug** | **Research**  **status** | **Indication** |
| --- | --- | --- | --- | --- | --- | --- | --- |
| 1 | TYMP | Thymidine phosphorylase | Angiogenesis | 13.4278/  48.9242 | Tipiracil | Approved | Metastatic colorectal cancer |
| 2 | A2M | Alpha-2-macroglobulin | Inflammation | 11.5790 | Bacitracin | Approved | Skin infections |
| 3 | GALK1 | Galactokinase | Metabolism | 5.8177 | Phosphoaminophospho-nic acid-Adenylate Ester | Experimental | Nutritional disease |
| 4 | GPX2 | Glutathione peroxidase 2 | DNA damage and repair | 3.7002 | Glutathione | Approved | Nutritional disease |
| 5 | PSMB8 | Proteasome subunit beta type-8 | Immunity | 3.6540 | Carfilzomib | Approved | Multiple myeloma |
| 6 | C1QB | Complement C1q subunit B subcomponent | Immunity | 3.2013 | Cetuximab | Approved | Metastatic colorectal  cancer |
| 7 | GNMT | Glycine N-methyltransferase | DNA damage and repair | 2.5773 | Ademetionine | Approved | Cofactor |
| 8 | APCS | Serum amyloid P-component | Inflammation | 2.5185 | Copper, Zinc, Calcium | Approved | Nutritional disease |
| 9 | ACE | Angiotensin-converting enzyme | Angiogenesis | 1.9703 | Captopril | Approved | Hypertension |
| 10 | CYP51A1 | Lanosterol 14-alpha demethylase | Metabolism | 1.9400 | Itraconazole | Approved | Fungal infections |
| 11 | OXCT1 | Succinyl-CoA:3-ketoacidco- enzyme A transferase 1 | Metabolism | 0.8043 | Succinic acid | Approved | Nutritional disease |
| 12 | GALE | UDP-glucose 4-epimerase | Metabolism | 0.6891 | UDP-alpha-D-glucuronic acid | Experimental |  |
| 13 | CXCL12 | Stromal cell-derived factor 1 | Immunity | 0.4807 | Tinzaparin | Approved | Venous thromboembolism |
| 15 | AKR1B1 | Aldo-keto reductase family1 member B1 | Inflammation | 47.6480 | Sulindac | Approved | Acute or chronic inflammatory conditions |
| 16 | PFN1 | Profilin-1 | Angiogenesis | 42.5952 | Dihydroartemisinin | Experimental | Malaria |
| 17 | ARPC1B | Actin-related protein 2/3 complex subunit 1B | Immunity | 29.9239 | N-[2-(2-methyl-1H-indol3yl)- ethyl]thiophene-  2-carboxamide | Experimental |  |
| 18 | LGALS9 | Galectin-9 | Immunity | 25.7911 | (R)-1-Para-Nitro-Phenyl-2-  Azido-ethanol | Experimental |  |
| 19 | GNPDA1 | Glucosamine-6-phosphate isomerase 1 | Metabolism | 23.9446 | Beta-D-Glucose | Experimental |  |
| 20 | VCP | Transitional endoplasmic reticulum ATPase | DNA damage and repair | 23.7186 | Phenethyl Isothiocyanate | Experimental |  |
| 21 | MYL12A/B | Myosin regulatory light chain 12A/B | Inflammation | 19.8024 | 4-[4-(2,5-dioxo-pyrrolidin-1- yl)-phenylamino]-4-hydroxy- butyric acid | Experimental |  |
| 22 | GAPDH | Glyceraldehyde-3-phosphate dehydrogenase | Metabolism | 18.8592 | NADH | Approved | Parkinson's disease, Alzheimer's disease and |
| 23 | GSTP1 | Glutathione S-transferase P | Metabolism | 18.5594 | Clomipramine | Approved | Obsessive-compulsive component |
| 24 | C1QC | Complement C1q subcomponent subunit C | Immunity | 17.9941 | Cetuximab | Approved | metastatic colorectal cancer |
| 25 | ITGAV | Integrin alpha-V | Angiogenesis | 17.7539 | Cilengitide | Investigational | Gliomas, Leukemia, and Lung Cancer |
| 26 | IGFBP7 | Insulin-like growth factor- binding protein 7 | Proliferation and invasion | 17.2963 | Insulin pork | Approved | Type I and II diabetes mellitus |
| 27 | NADSYN1 | Glutamine-dependent NAD(+)synthetase | Metabolism | 17.0255 | L-Glutamine | Approved | Acute complications of sickle cell disease |
| 28 | SLC25A20 | Mitochondrial protein carnitine/acylcarnitine carrier | Metabolism | 16.1767 | Levocarnitine | Approved | Primary systemic carnitine deficiency |
| 29 | VWF | von Willebrand factor | Angiogenesis | 16.0179 | Caplacizumab | Approved | Acquired thrombotic thrombocytopenic purpura |
| 30 | BCAT2 | Branched-chain-amino-acid aminotransferase | Metabolism | 14.7494 | Pyridoxal phosphate | Approved | Nutritional disease |
| 31 | S100A11 | Protein S100-A11 | Inflammation | 12.2259 | Phosphonothreonine | Experimental |  |
| 32 | GPX3 | Glutathione peroxidase 3 | DNA damage and repair | 11.3056 | Glutathione | Approved | Nutritional disease |
| 33 | VCAN | Versican core protein | Angiogenesis | 9.3562 | Hyaluronic acid | Approved | Osteoarthritis |
| 34 | NRP1 | Neuropilin-1 | Angiogenesis | 8.6977 | Palifermin | Approved | Oral Mucositis |
| 35 | COL3A1 | Collagen alpha-1(III) chain | Proliferation and invasion | 7.1835 | Collagenase clostridium histolyticum | Approved | Dupuytren's contracture， Peyronie's disease |
| 36 | CASP1 | Caspase-1 | Inflammation | 2.9691 | Aspirin | Approved | Pain and fever，myocardial infarction |
| 37 | PTGIS | Prostacyclin synthase | Inflammation | 2.6049 | Phenylbutazone | Approved | Backache and ankylosing spondylitis |
| 38 | PSMB9 | Proteasome subunit beta type-9 | Immunity | 1.0221 | Carfilzomib | Approved | Multiple myeloma |
| 39 | ACACB | Acetyl-CoA carboxylase 2 | Metabolism | 0.4337 | Biotin | Approved | Nutritional disease/multiple sclerosis |

Relationship with the occrrence and progression of HCC: ^1-14^ Proteins related to PME-O,^15-39^ proteins related to PME-P; Drug Category: ^30,34-35^ drugs targeting the proteins were biological macromolecule, of which ^30^drugs were monoclonal antibody, ^34-35^ drugs were recombinant protein, others were small molecule compounds；Risk coefficient was the weight of proteins in the occurrence or progression of HCC；HCC: hepatocellular carcinoma.

**Table S8 Primer sequences**

| Primers for real-time RT-PCR | | |
| --- | --- | --- |
| Mapk1 | S | GGTTGTTCCCAAATGCTGACTC |
|  | A | ATGGGCTCATCACTTGGGTCA |
| Nfkb3 (Rela) | S | CGAGTCTCCATGCAGCTACG |
|  | A | TTTCGGGTAGGCACAGCAATA |
| Rsk1 | S | CTACATTATGCCTTCCAGACCGAG |
|  | A | CTGTCCCGCAGAAGGAGTAAGC |
| Rsk2 | S | CATGGAGTTTGCCGTGAAGATTA |
|  | A | ATCTTATCCAGCAATTCACCTCC |
| Hif1α | S | TTGCTTTGATGTGGATAGCGATA |
|  | A | CATACTTGGAGGGCTTGGAGAAT |
| Vegf | S | GAGCGTTCACTGTGAGCCTTGT |
|  | A | TTAACTCAAGCTGCCTCGCCT |
| TYMP (human) | S | CTTGTGGACAAGCATTCCAC |
|  | A | TCACATCTCTGGCTGCATATAG |
| Tymp (mouse) | S | GCACAATGCTTTAGCTAGGC |
|  | A | CATTCTTTGGATTAGCGGACAG |
